# Conspiracy Beliefs, Institutional Mistrust, and Health‐Related Behaviours During the COVID‐19 Pandemic in Burkina Faso: A Mediation Analysis

**DOI:** 10.1002/hpm.70058

**Published:** 2026-01-28

**Authors:** Gabin F. Morillon, Marlène Guillon, Jacky Mathonnat

**Affiliations:** ^1^ Université de Montpellier Montpellier Recherche en Economie Montpellier France; ^2^ Fondation pour les Etudes et Recherches sur le Développement International (FERDI) Clermont‐Ferrand France; ^3^ Université Clermont Auvergne Centre d’Etudes et de Recherches sur le Développement International (CERDI) and Fondation pour les Etudes et Recherches sur le Développement International (FERDI) Clermont‐Ferrand France

**Keywords:** Burkina‐Faso, conspiracy beliefs, COVID‐19, health behaviours, mediation analysis, structural equation modelling, trust

## Abstract

**Background:**

No study has yet examined the conjoint role of institutional trust and COVID‐19 conspiracy beliefs on compliance with COVID‐19 preventive behaviours among populations of African countries. This study aims to deepen the understanding of the relationship between institutional mistrust, conspiracy beliefs, and health‐related behaviours in the context of an African country during the COVID‐19 pandemic.

**Methods:**

A cross‐sectional research design was employed, and a paper‐based survey using face‐to‐face interviews was conducted among the general adult population of Burkina Faso, collecting data on institutional mistrust, conspiracy beliefs, health‐related behaviours (i.e., vaccination attitudes, agreement to sanitary measures, and containment‐related behaviours), information‐seeking behaviours (including traditional and digital sources of information), and negative sentiments. We defined a conceptual model according to the existing literature. A mediation analysis was employed to examine the direct effect of institutional mistrust on health‐related behaviours and its indirect effect through conspiracy beliefs endorsement. Additionally, the effects of information‐seeking behaviours and negative sentiments on mistrust and conspiracy beliefs were explored.

**Results:**

We find that institutional mistrust had a direct negative effect on all health‐related behaviours and an indirect effect through conspiracy beliefs which themselves had a direct negative effect on protective behaviours. The partial mediation accounted for 12% (agreement with sanitary measures) to 34% (vaccination attitudes) of the total effect of institutional mistrust on health‐related behaviours. Information seeking on traditional media and negative sentiments had positive effects on conspiracy beliefs and institutional mistrust, respectively.

**Conclusions:**

Our findings underscore the need for medium‐to long‐term policies aimed at restoring and perpetuating trust in institutions and curbing conspiracy beliefs for fighting future pandemics. In short term, health promotion campaigns should be channelled through the sources of information in which individuals have the greatest confidence such as religious and traditional leaders.

## Highlights

- Conspiracy beliefs and institutional mistrust negatively impact health behaviours.
- Institutional mistrust indirectly affects health behaviours via conspiracy beliefs.
- COVID‐19 negative sentiments heighten institutional mistrust and traditional information seeking fosters conspiracy beliefs endorsement.

## 1 Introduction

Through being less affected than the rest of the world by the successive COVID‐19 epidemic waves, Sub‐Saharan (SSA) countries have implemented very strict COVID‐19 containment measures since the beginning of the epidemic. While of crucial importance in preventing a surge of the epidemic, the drastic containment measures adopted by most SSA countries have heavily impacted firms, plunged their economies, and pushed a significant part of their populations into poverty given the importance of informal employment and the absence of social safety nets [1, 2, 3]. Burkina Faso, which falls into the category of Least Developed Countries and in which 40% of the population lives below the national poverty line, is no exception among SSA countries regarding its severe response to the pandemic. The first case of COVID‐19 was reported in Burkina Faso on March 9, 2020. As soon as the first cases appeared, the Burkinabe government communicated widely about the risks of the pandemic and took drastic containment measures (e.g., lockdown, closure of international borders and of non‐essential businesses) to prevent its rapid spread. As a result, on August 1st, 2021, at the end of the survey on which this study is based, the total number of cumulative confirmed cases was only 1143, including 170 deaths, concentrated in urban areas. Nevertheless, the sanitary crisis, government measures and imported recession led to a fall of the real GDP growth rate of the country from 5.7% in 2019 to 1.9% in 2020, followed by an estimated rebound to 6.9% in 2021^1^.

In the context of the emergence of a new infectious disease about which little was known early on regarding transmission modes or means of protection, studies conducted in numerous countries have demonstrated the crucial importance of trust in the adoption of the recommended COVID‐19 preventive behaviours. This specific context was also prone to the development and diffusion of various COVID‐19 misinformation on traditional and social media and false or conspiracy beliefs on COVID‐19 were documented as barriers to the uptake of COVID‐19 preventive behaviours around the world (see Section 2 for details on the literature available). However, to the best of our knowledge, no study has yet examined the conjoint role of both trust and COVID‐19 conspiracy beliefs on compliance with COVID‐19 preventive behaviours among populations of African countries. Indeed, studies which assessed the drivers and barriers of COVID‐19 preventive behaviours in SSA countries mainly focused on socioeconomic and demographic factors and on COVID‐19 and preventive behaviours' perceptions [4, 5, 6, 7, 8, 9, 10, 11, 12, 13, 14, 15, 16, 17, 18, 19, 20, 21].

This study employed a cross‐sectional research design, and a paper‐based survey was conducted in Summer 2021 among a large sample of the population in Burkina Faso. Our first objective is to study how trust and conspiracy beliefs directly influence COVID‐19 preventive behaviours. Insofar as low trust in institutions can be a breeding ground for the sensitivity to misinformation and for the development of conspiracy beliefs, we also investigate the indirect impact of trust on preventive behaviours through the adherence to conspiracy beliefs using a mediation analysis. With the goal to provide the most comprehensive model of behaviour adoption, and to derive concrete policy implications from our results, our second objective is to explore the antecedents of trust and conspiracy beliefs. Our study contributes both to the literature on the factors associated with COVID‐19 health‐related outcomes in Sub‐Saharan Africa and to the literature that explores the relationships between mistrust, conspiracy beliefs, and COVID‐19 health‐related outcomes.

The structure of our work is organised as follows. Section 2 outlines the hypotheses that we tested in our study, establishing a solid theoretical foundation for our research. In Section 3, we present the methodology employed, including details on the cross‐sectional design and the survey method, measurements utilised, and the analyses conducted using structural equations modelling (SEM). Section 4 presents the primary results obtained from the analyses, shedding light on the intricate connections between health‐related outcomes, conspiracy beliefs, and institutional mistrust. Finally, in Section 5, we conclude by summarising the main findings and their contribution to the existing literature, discussing their policy implications, and critically evaluating the strengths and limitations of our approach.

## 2 Hypotheses

This section presents the hypotheses we formulated and the resulting model that was tested in the empirical analysis. All references are supplied in Online Supporting Information S1: File 1.

Previous research has shown that COVID‐19 vaccine hesitancy, containment‐related behaviours, and agreements with sanitary measures are strongly influenced by a lack of trust in governments, health authorities, healthcare institutions, the pharmaceutical industry, and science [22, 23, 24, 25, 26, 27, 28, 29, 30, 31, 32, 33, 34, 35, 36, 37, 38, 39]. This trend has been observed in all regions of the world. Trust in scientists, government, and others was found to have a significant positive impact on adherence to non‐pharmaceutical interventions in a large survey spanning 12 countries [22]. Similar positive relationships between trust and vaccination intention or behaviours were found in the United States [23, 24], China [25, 26], Japan [27], European countries [24, 28, 29, 30, 31], Latin American and Caribbean countries [32, 33], and Arab countries [34, 35]. Among african countries, recent studies conducted in South Africa, Nigeria, and Ghana have found that obstacles to COVID‐19 vaccination acceptance included political discontent [36], low trust in government, and public health authorities or vaccines developers [37, 38]. Finally, a worldwide based systematic review reported that trust in government was a significant predictor of vaccination acceptance [39]. Based on these findings, we hypothesise that mistrust has a direct negative effect on the adoption of COVID‐19 preventive behaviours.

H 1 Mistrust in institutions has a direct negative effect on COVID‐19 containment‐related behaviours, vaccination attitudes, and sanitary measures agreement.

The rapid development of COVID‐19 vaccines in response to a new infectious disease has created an ‘infodemic’ context in which misinformation about COVID‐19 and vaccines spread quickly through traditional and social media. False and conspiracy beliefs about COVID‐19 were negatively associated with vaccination uptake or intention in studies conducted across North America [40, 41], Europe [28, 30, 42], Asia [43, 44, 45], Latin America [33], and the Arab world [35, 46]. In SSA countries, infertility rumours [14] or false information about the COVID‐19 vaccines [11] were identified as barriers to the COVID‐19 vaccination campaigns in Nigeria and Cameroon. Self‐reported adherence to recommended behaviours against COVID‐19 was also found to be negatively related to conspiracy beliefs in various studies. In Germany, conspiracy mentality was negatively associated with health‐protective behaviours, including adherence to rules such as wearing face masks and reducing physical contacts [47]. In the United Kingdom, COVID‐19 conspiracy belief endorsement was negatively related to acceptance of preventive measures and health‐related behaviours [48]. Meanwhile, in the United States, endorsement of general conspiracy beliefs and COVID‐19 conspiracy theories were positively related to attitudes towards easing restrictions [49]. Following the literature, we hypothesise that adherence to conspiracy beliefs have a direct negative impact on the adoption of COVID‐19 preventive behaviours.

H 2 The adherence to COVID‐19 conspiracy beliefs has a direct negative impact on COVID‐19 containment‐related behaviours, vaccination attitudes, and sanitary measures agreement.

The field of social psychology has long demonstrated that individuals with low levels of trust, whether it be in general [50], in the government [51], in neighbours, or in relatives [52], are more susceptible to misinformation and conspiracy theories. Recent research conducted during the COVID‐19 pandemic has confirmed this relationship. In Germany, Bruder & Kunert (2022) [53] found that less trust in official information sources like the Public Health Ministry, the Robert Koch Institute, or the World Health Organization (WHO) was associated with a tendency to believe in vaccine‐related conspiracies. A study conducted in Denmark and Germany also found that institutional trust was negatively related to political COVID‐19 conspiracies [54]. Trust in the government to provide coronavirus‐related information in South Korea was also negatively related to conspiracy beliefs [55]. Lastly, a study conducted in the United Kingdom, Ireland, and Spain found that not all sources of trust were equally related to conspiratorial behaviours, with trust in politics having a much greater effect than trust in scientists or legal institutions [56]. Overall, available literature shows that low levels of trust in institutions can create a fertile ground for the spread of misinformation and the development of conspiracy beliefs. Then, our hypothesis is that institutional mistrust has a direct positive effect on the tendency to believe in COVID‐19 conspiracy theories.

H 3 Institutional mistrust is associated with increased tendency to believe in COVID‐19 conspiracy theories.

If mistrust has a direct effect on both COVID‐19 preventive behaviours (H2) and COVID‐19 conspiracy theories (H2), we can formulate the following hypothesis.

H 4 Institutional mistrust has an indirect negative effect on the adoption of COVID‐19 containment‐related behaviours/vaccination intention/sanitary measures agreement through the increased tendency to believe in COVID‐19 conspiracy theories.

While conspiracy thinking is disproportionately shared online, with false news spreading farther and faster than true news on platforms like Twitter [57], traditional media outlets such as radio, television, and newspapers are more likely to provide verified information, reducing individuals' exposure to COVID‐19 misinformation. Then, the association between information seeking and conspiracy belief endorsement remains unclear [58] and previous research has demonstrated the ambivalent relationship between information seeking and COVID‐19 conspiracy beliefs, depending on the type of media used. In high‐income countries, exposure to traditional and digital media has been positively and negatively associated with both generic and COVID‐19‐specific conspiracy beliefs, respectively [59, 60, 61, 62, 63]. We then formulate the following hypothesis.

H 5 Increased digital and traditional information seeking is associated with higher (H5a) and lower (H5b) beliefs in conspiracy theories, respectively.

The association between information seeking and institutional (mis)trust might also be dependent on the type of media used. A recent study conducted in the Netherlands found that greater diversity in media use was linked to increased trust in institutions while non‐print‐oriented users, particularly those who used Facebook frequently, exhibited lower levels of institutional trust [64]. Several studies conducted in China also demonstrated that while traditional media use positively impacted institutional trust, social media use had a negative effect on trust in institutions [65, 66, 67, 68]. Similar results were found in Ghana [69]. However, within traditional or digital media, results might also diverge depending on the precise type of news platform used. In Sweden, Strömbäck et al. (2016) [70] found that there was a nuanced positive relationship between news media use and political trust, with public or commercial TV news having diverging effects. In a cross‐sectional study across 27 European countries, Ceron (2015) [71] found that websites and social media had positive and negative relationships with political trust, respectively. Based on the literature available, we express the following hypothesis.

H 6 Information seeking on traditional and digital media are negatively ($H_{6a}$) and positively ($H_{6b}$) related to institutional mistrust.

Before the pandemic, studies found a negative relationship between media use and anxiety and depression [72]. The negative association between media use and negative sentiments was confirmed in the COVID‐19 context. A study conducted in the United States found that COVID‐19 information seeking frequency on news television channels and social media had a positive effect on emotional distress measured by overwhelm, fear, and anxiety [73]. In Turkey, Akca and Ayaz‐Alkaya (2022) [74] also found a negative relationship between the frequency of COVID‐19 information seeking in media tools and negative emotions or perceived stress. In South Korea, information seeking frequency was furthermore found to be negatively related to anxiety and fear of COVID‐19 [75]. We then hypothesise that increased information seeking on traditional and digital media are associated with higher negative sentiments regarding the COVID‐19 pandemic.

H 7 Increased traditional (H7a) and digital information (H7b) seeking are associated with higher negative sentiments regarding the COVID‐19 pandemic.

Research in social psychology consistently found that conspiracy beliefs were associated with negative emotions [76], particularly feelings of powerlessness and anxiety [58]. Individuals with emotion dysregulation were also more likely to adopt generic or specific conspiratorial beliefs as a means of feeling in control and secure [77]. In the COVID‐19 context, a study conducted in South Korea found that COVID‐19 conspiracy beliefs were positively associated with the perceived risk of disease and anxiety [55]. Therefore, we hypothesise that individuals who experience more negative emotions during the COVID‐19 pandemic are more likely to believe in COVID‐19 conspiracy theories.

H 8 Negative sentiments during the sanitary crisis are associated with increased beliefs in COVID‐19 conspiracy theories.

Considering individuals' emotions is crucial for building trust. Before the pandemic, the literature highlighted the association between emotions and trust. Dunn and Schweitzer (2005) [78] studied the effect of four emotions, characterised by either positive or negative valence and by either appraisals of other‐person control (anger and gratitude) or appraisals of personal control (pride and guilt), on trust. They found that negative emotions decreased trust and that among emotions with the same valence those with other‐person control influenced trust to a greater extent. Using an experiment, Myers and Tingley (2017) [79] also found that negative emotions associated with uncertainty, in particular anxiety, had a negative impact on interpersonal trust measured by the trust game. We then hypothesise that negative sentiments during the sanitary crisis are associated with increased institutional mistrust.

H 9 Negative sentiments during the sanitary crisis are associated with increased institutional mistrust.

## 3 Methodology

### 3.1 Cross‐Sectional Research Design

The sample is composed of French‐speaking adults living in Burkina Faso. The recruitment of a sample of 1000 Burkinabe respondents was operated by an independent survey company (OBAAS CONSULTING) during Summer 2021 using 26 strata from the 2019 population distribution estimates by the National Institute of Demographic Studies of Burkina Faso and representing rural and urban areas in the 13 Burkinabe regions. The private firm in charge of the survey used for this study a representative sample of 1000 households originally constructed and applied by the Burkinabe National Institute of Statistics and Demography (INSD). This includes the INSD random selection of Enumeration Areas and a full three‐stage sampling procedure. A sample of individuals was drawn from each of the 26 strata. In the first stage, Enumeration Areas (EA) from the national census were randomly selected within each stratum. In the second stage, a sample of 12 households was randomly drawn from each EA. In the third stage, one individual from each sampled household was selected to meet predefined quotas for age, gender, occupation, and ethnicity.

### 3.2 Ethics

This study was approved by the ethics committee of the University of Montpellier under number UM 2023‐003. The academic purposes of the study were presented to the respondents before asking whether they agreed to participate. Eligibility criteria included being over 18 years and living in Burkina Faso. Data received from the survey company and used in the analysis are strictly anonymous.

### 3.3 Measures

#### 3.3.1 Study Outcomes

##### 3.3.1.1 COVID‐19 Vaccination Attitudes

**FIGURE 1:**
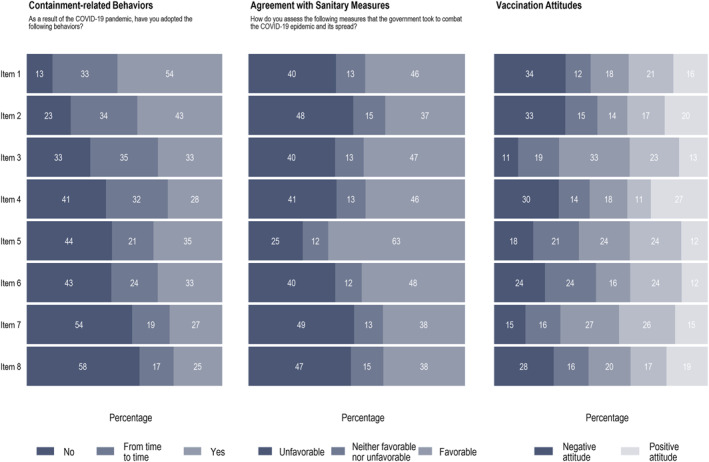
Containment‐related behaviours, agreement with sanitary measures, and vaccination attitudes.

COVID‐19 vaccination attitudes were measured using an 8‐item questionnaire designed for the study based on previous literature [80, 81]. A 5‐point Likert scale was used for each item. Items were: (1) *If a vaccine against COVID‐19 was offered to you for free today, would you accept to be vaccinated*? (from *I would not accept at all* to *I would certainly accept*), (2) *When the vaccine against COVID‐19 will be widely available and free of charge in Burkina Faso:* (from *I will get vaccinated as soon as possible* to *I will not be vaccinated*), (3) *With regard to vaccination against COVID‐19 I would describe my attitude as:* (from *Very negative* to *Very positive*), (4) *If the COVID‐19 was available at the local health centre, doctor's office, or pharmacy:* (from *I would get vaccinated as soon as possible* to *I would never get vaccinated*), (5) *If members of my family or friends were considering being vaccinated against COVID‐19:* (from *I would suggest them not to get vaccinated* to *I would encourage them to get vaccinated*), (6) *I would describe myself as:* (from *Looking forward to getting vaccinated against COVID‐19* to *Anti‐vaccination COVID‐19*), (7) *Getting vaccinated against COVID‐19 is:* (from *Very important* to *Really irrelevant*), and (8) *If I have children (or if I had children):* (from *I will definitively have them vaccinated against COVID‐19* to *I will definitively not vaccinate them against COVID‐19*). Cronbach's *α* was equal to 0.956 (Figure 1, Online Supporting Information S1: File 4).

##### 3.3.1.2 Containment‐Related Behaviour (CRB)

Respondents were asked *As a result of the COVID‐19 pandemic, have you adopted the following behaviours?* on a 3‐point scale (no [0], from time to time [1], yes [2]). Items were: (1) *You wash your hands more often and/or for longer*, (2) *Coughing or sneezing into your elbow or a handkerchief*, (3) *You have stopped shaking hands in greeting*, (4) *You keep a distance of one meter from other people*, (5) *You have reduced your travels*, (6) *You avoid crowded places (public transport, places of worship, wedding ceremony, baptism,* etc.), (7) *You have cut down on visits to your family and friends*, and (8) *You have reduced physical contacts with other members of your household*. Cronbach's *α* was equal to 0.931 (Figure 1, Online Supporting Information S1: File 4).

##### 3.3.1.3 COVID‐19 Agreement With Preventive Measures

We asked respondents *How do you assess the following measures that the government took to combat the COVID‐19 epidemic and its spread?*. A total of eight measures were included: (1) closure of schools and universities, (2) closure of places of worship, 3. closure of non‐essential shops (bars, non‐food and health shops, etc.), (4) introduction of a curfew and movement control by the police, gendarmerie, and army, (5) introduction of mandatory medical checks and quarantine for people entering Burkina Faso, (6) closure of Burkina Faso's borders, (7) general lockdown with a ban on leaving the home (except for medical reasons and food purchase), and 8. closure of all non‐essential businesses and institutions. Each item was assessed with three modalities: unfavourable (0), neither favourable nor unfavourable (1), and favourable (2). Cronbach's *α* was equal to 0.941 (Figure 1, Online Supporting Information S1: File 4).

#### 3.3.2 Variables of Interest

##### 3.3.2.1 COVID‐19 Conspiracy Beliefs

**FIGURE 2:**
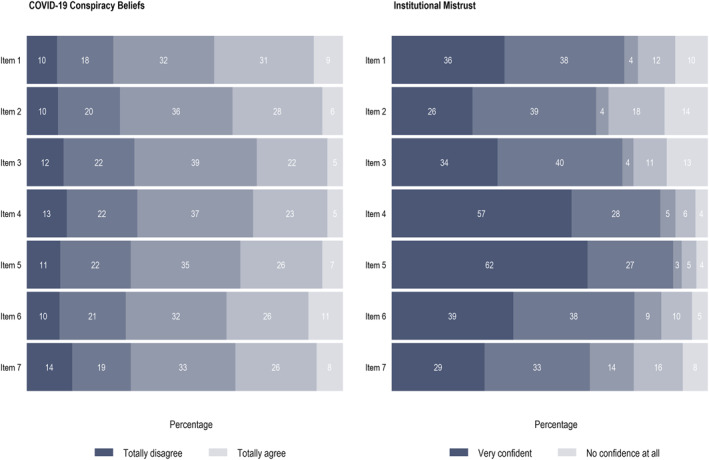
COVID‐19 conspiracy beliefs and institutional mistrust.

Based on previous literature [42, 61, 82], COVID‐19 conspiracy beliefs were assessed using seven items rated on a 5‐point Likert scale ranging from 1 (Totally disagree) to 5 (Totally agree). Items were: (1) *Government withholds important information on the COVID‐19 pandemic from the Burkinabe population*, (2) *Doctors and scientists hide important information from the population on the COVID‐19 epidemic*, (3) *The COVID‐19 epidemic is a deliberate attempt to reduce the population size of poor countries*, (4) *The COVID‐19 virus is intentionally presented as dangerous to manipulate the public*, (5) *The COVID‐19 epidemic is part of a global effort to impose mandatory vaccination*, (6) *Pharmaceutical industry promotes the spread of the COVID‐19 to make money*, and (7) *The COVID‐19 pandemic is a deliberate attempt by rich country governments to better control the populations of poor countries*. Cronbach's *α* was equal to 0.919 (Figure 2, Online Supporting Information S1: File 4).

##### 3.3.2.2 Institutional Mistrust

Institutional mistrust was measured using seven items assessing trusts in (1) the current President, (2) the government, (3) local authorities (municipal/regional councillors, mayors, prefects, etc.), (4) traditional authorities (neighbourhood chief, village/canton chief, etc.), (5) religious authorities (religious leader, etc.), (6) scientific authorities (doctors, other health professionals, researchers, etc.), and (7) the World Health Organization. Each item was rated on a 5‐point Likert scale ranging from 1 (Very confident) to 5 (No confidence at all). Cronbach's *α* was equal to 0.841 (Figure 2, Online Supporting Information S1: File 4).

##### 3.3.2.3 COVID‐19 Negative Sentiments

Three items were used to assess negative sentiments about the COVID‐19 pandemic: *‘When you think about the situation with COVID‐19 in Burkina Faso, do you feel fear/hope/angry?’*. Satisfaction with life was reversed and added to those three items to create a negative sentiments score. Each item was scaled between 0 and 1 and summed to obtain a score ranging from 0 (positive sentiments) to 4 (negative sentiments). Cronbach's *α* was equal to 0.360 (Online Supporting Information S1: File 4).

##### 3.3.2.4 Information Seeking Frequency

We asked how often respondents looked for information on different traditional media (i.e., radio, television, print press) and digital media (i.e., social networks, websites). Frequency was rated on a 5‐point Likert scale ranging from 0 (Never) to 4 (Everyday) and items were summed to obtain a traditional and digital media score (Online Supporting Information S1: File 4).

#### 3.3.3 Sociodemographic and Socioeconomic Background

Age, gender, type of residency, and educational level were found to be significant predictors of vaccination and adherence to containment‐related behaviours worldwide [39, 83]. Thus, to limit the omitted variable bias, all analyses include controls for a set of demographic and socioeconomic variables: gender, age, school attendance (yes; no), marital status (live alone; live with a partner), work status (two dummy variables for self‐employment and salaried employment), and type of residency (rural; urban).

### 3.4 Analysis

#### 3.4.1 Descriptive Analysis

Continuous variables, and binary or ordered categorial variables were described using mean, standard deviation, median, and range. Pearson *r* coefficients between continuous variables were also reported.

The internal consistency of each latent variable was assessed using Cronbach's *α*. Item‐test correlation, item‐rest correlation, inter‐item correlation, and the value of the Cronbach's *α* if an item is removed were also reported. Each item was also described with t‐tests to assess the equality with the middle value and with pairwise Pearson's r correlation coefficients using Bonferroni adjustment to calculate significance levels. All statistics are available in Online Supporting Information S1: File 4.

Bivariate analyses were also performed between the COVID‐19 Vaccine Attitudes, the Agreement with Sanitary Measures, the Containment‐related Behaviours, the Conspiracy Beliefs, and the Institutional Mistrust latent variables and independent/control variables. Relevant tests were performed according to the nature and distribution of the variables (i.e., one‐way analysis of variance, Kruskal‐Wallis H test, and Bartlett's test for equality of variances). A *p*‐value less than 0.05 was considered as statistically significant and all p‐values were reported.

#### 3.4.2 Structural Equation Modelling

A Structural Equations Model (SEM) is a statistical tool enabling the analysis of complex interrelationships among various variables in a system [84]. It permits researchers to simultaneously explore both direct and indirect effects among multiple variables. This method involves constructing a theoretical model based on theory or existing knowledge from literature, illustrating the interconnectedness of different variables [85]. These variables can represent diverse concepts or constructs, such as attitudes or behaviours in the domain of health. Within SEM, two fundamental components are the measurement model and the structural model. The measurement model assesses the quality of the relationship between observed variables and the latent constructs they represent (in our case, health outcomes, adherence to conspiracy beliefs, and mistrust in institutions). This evaluation ensures the accuracy of measurements by employing techniques like factor analysis. On the other hand, the structural model examines the relationships between latent variables themselves and between latent and observed variables. It focuses on uncovering how these constructs interact, highlighting relationships between them. SEM are assessed by using observed data to estimate the relationships between variables to reveal both direct and indirect relationships, often named mediation effects, occurring through intermediate variables (see Online Supporting Information S1: File 3). SEM facilitates the examination of how different economic, social, and health‐related factors interact and impact health outcomes. SEM allows to test hypotheses, validate theoretical models, and disentangle the underlying mechanisms driving relationships between variables. The dual examination of the measurement model and structural model within SEM deepens our understanding of the accuracy of measurements and the underlying relationships between concepts, providing a comprehensive framework to highlight the interplay among various factors.

Based on Lin et al. (2017) [86] and Hooper et al. (2007) [87], several absolute and parsimony fit indices were reported to assess models' fit. Although model Chi‐square ($\chi^{2}$) assumes multivariate normality and is sensitive to sample size, it is commonly accepted to report its significance. The root mean square error of approximation (RMSEA) with its confidence interval was reported as recommended. A cut‐off value close to 0.05 was considered. The standardized root mean square residual (SRMR) was considered for its meaningfulness. A value less than 0.05 indicates a good fit. Note that a higher number of parameters tends to decrease the SRMR. The Comparative fit index (CFI) is a revised form, adjusted for the sample size, of the Normed‐fit index (NFI) which compared the $\chi^{2}$ of the model to the $\chi^{2}$ of the null model. The value ranges between 0 and 1, a value close to 1 indicating a good fit. A cut‐off of 0.95 was retained. Finally, the log‐likelihood function and the number of parameters were also reported.

All analysis were performed using Stata 17 (StataCorp. 2021. *Stata Statistical Software: Release 17*. College Station, TX: StataCorp LLC.). Structural equation modelling was performed using the ‐sem‐ command. The mediation effect was computed using the ‐medsem‐ command with the Zhao, Lynch, and Chen's approach [88]. Control variables were specified as continuous or dichotomous.

**FIGURE 3:**
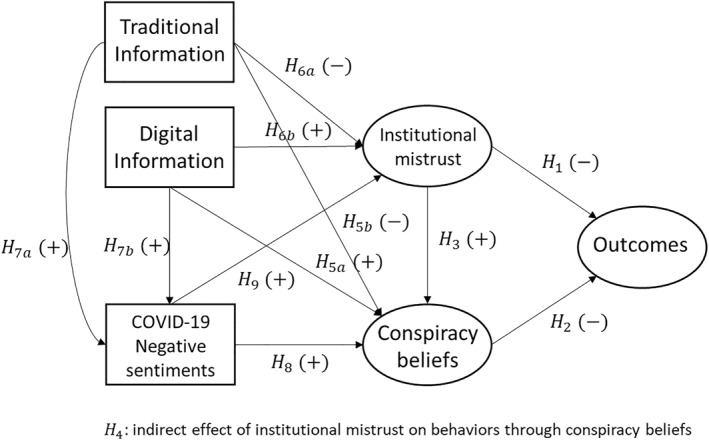
Structural equation modelling.

Based on hypotheses H1 to H9, the conceptual model represented in Figure 3 was tested. For clarity Figure 3 only depicts variables of interest included in the analysis. All control variables are nevertheless used as covariates within the SEM analysis. Three outcomes were used: COVID‐19 vaccination attitudes, agreement with sanitary measures, and containment‐related behaviours. Variables in circles are considered as latent.

## 4 Results

### 4.1 Population Descriptive Analysis

Compared to the general population of Burkina Faso, our sample was skewed towards men and urban residents since only 35.38% of our respondents were female and only 39.68% lived in urban areas (Table 1). While efforts were made to meet the socioeconomic and demographic quotas, the final sample is not fully representative of the Burkinabe population by sex and place of residency (female and rural residents being underrepresented) due to cultural issues and to difficulties in data collection related to insecurity at the time of the survey in the Sahel, North, and East regions. The average age was 37 (± 12.76) and 71.70% declared living alone. More than 60% of respondents attended school, while 24% held salaried employment, and 29% identified as self‐employed (Table 1).

**TABLE 1:** Sociodemographic and ‐economic descriptive statistics.

	*N*	Mean	SD	Range	Frequency	2019 population census (*N* = 20,505,155)
Containment related behaviours	915	7.827	5.478	(0–16)		
Agreement with sanitary measures	917	8.336	6.219	(0–16)		
Vaccination attitudes	974	23.078	9.896	(8–40)		
Institutional mistrust	994	14.830	6.171	(7–35)		
Conspiracy beliefs score	998	20.827	6.299	(7–35)		
Traditional information seeking	987	6.342	2.882	(0–12)		
Digital information seeking	993	2.879	2.977	(0–8)		
Negative sentiments	976	2.513	0.462	(1–4)		
Gender						
Male	642				64.20%	9,900,847 (48.28%)
Female	358				35.80%	10,604,308 (51.72%)
Ratio M/F	0.558					1.07
Age (in years)	1000	36.982	12.755	(18–92)		
Attended school						
Yes	633				63.36%	
No	366				36.64%	
Marital status						
Live alone	717				71.70%	
Live with a partner	283				28.30%	
Salaried employment						
Yes	239				23.90%	
No	761				76.10%	
Self‐employed						
Yes	291				29.19%	
No	706				70.81%	
Type of residency						
Rural	602				60.32%	15,145,043 (73.86%)
Urban	396				39.68%	5,360,112 (26.14%)
Region of residency						
Boucle du Mouhoun	93				9.30%	1,901,269 (9.27%)
Cascades	38				3.80%	812,466 (3.96%)
Centre	217				21.70%	3,030,384 (14.78%)
Centre‐Est	81				8.10%	1,580,508 (7.71%)
Centre‐Nord	67				6.70%	1,874,669 (9.14%)
Centre‐Ouest	93				9.30%	1,660,135 (8.10%)
Centre‐Sud	35				3.50%	788,731 (3.85%)
Est	68				6.80%	1,942,805 (9.47%)
Hauts‐Bassins	126				12.60%	2,239,840 (10.92%)
Nord	35				3.50%	1,722,115 (8.40%)
Plateau central	46				4.60%	978,614 (4.77%)
Sahel	61				6.10%	1,098,177 (5.36%)
Sud‐Ouest	40				4.00%	875,442 (4.27%)

Our sample exhibited a moderate adherence to COVID‐19–related health behaviours with mean scores for Containment‐related Behaviours, Agreement with Sanitary Measures, and Vaccination Attitudes equal to 7.827/16 (± 5.478), 8.336/16 (± 6.219), and 23.078/40 (± 9.896), respectively. It also exhibited a relatively high level of institutional mistrust (14.830/35 [± 6.171]) along with a relatively high endorsement of COVID‐19 conspiracy beliefs (20.827/35 [± 6.299]). Information seeking through traditional channels was relatively moderate (6.342/12 [± 2.882]), whereas information seeking through digital channels was low (2.879/8 [± 2.977]). Finally, participants showed an average level of COVID‐19 negative sentiments (2.513/4 [± 0.462]). Bivariate analysis revealed significant differences between almost all control variables and outcomes (i.e., Containment‐related Behaviours, Agreement with Sanitary Measures, and Vaccination Attitudes) and variables of interest (i.e., Conspiracy Beliefs and Institutional Mistrust) (See Online Supporting Information S1: File 2 for details).

### 4.2 Structural Equation Modelling

The three models exhibited a good fit. For containment‐related behaviours the RMSEA, the SRMR, and CFI were equal to 0.047 [0.044, 0.050], 0.043, and 0.941, respectively. Regarding vaccination attitudes, the corresponding values were 0.046 [0.043, 0.049], 0.040, and 0.952. Similarly, for agreement with sanitary measures, the values were 0.057 [0.054, 0.060], 0.045, and 0.922.

**FIGURE 4:**
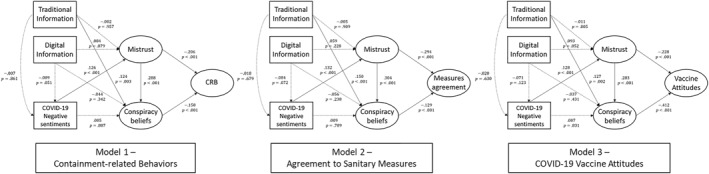
Structural Equations Models. CRB: Containment‐related behaviours, dashed lines represent non‐significant results at p=0.05.

Findings of the SEM for models 1 to 3 are depicted in Table 2 and Figure 4. Institutional mistrust and conspiracy beliefs had a significant negative impact on containment‐related behaviours, vaccine attitudes, and agreement with sanitary measures (*p* < 0.001 in all cases). Thus, both mistrust in institutions (H1) and the adherence to COVID‐19 conspiracy beliefs (H2) had a direct negative impact on all outcomes. Hypotheses 1 and 2 were then confirmed for all models. Furthermore, institutional mistrust positively influenced conspiracy beliefs (*p* < 0.001), confirming hypothesis H3. We found a partial mediation that accounted for 18.1%, 11.8%, and 33.9% of the total effect of institutional mistrust on containment‐related behaviours, agreement with sanitary measures, and vaccine attitudes (*p* < 0.001), respectively, thus supporting H4 (See Online Supporting Information S1: File 5 for details).

**TABLE 2:** Results of the structural equation models.

Structural equation modelling results						
Variables	Containment‐related behaviours		Agreement with sanitary measures		COVID‐19 vaccination attitudes	
	Standardized coefficient	p‐value	Standardized coefficient	p‐value	Standardized coefficient	p‐value
Containment‐related behaviours/Agreement with sanitary measures/Vaccination attitudes						
Institutional mistrust	**−0.206**	**< 0.001**	**−0.294**	**< 0.001**	**−0.228**	**< 0.001**
COVID‐19 conspiracy beliefs	**−0.158**	**< 0.001**	**−0.129**	**< 0.001**	**−0.412**	**< 0.001**
Institutional mistrust						
Traditional information seeking frequency score	−0.002	0.957	−0.005	0.909	−0.011	0.805
Digital information seeking frequency score	0.084	0.079	0.059	0.228	0.093	0.052
COVID‐19 negative sentiments score	**0.126**	**< 0.001**	**0.132**	**< 0.001**	**0.128**	**< 0.001**
COVID‐19 conspiracy beliefs						
Institutional mistrust	**0.288**	**< 0.001**	**0.304**	**< 0.001**	**0.283**	**< 0.001**
Traditional information seeking frequency score	**0.124**	**0.003**	**0.150**	**< 0.001**	**0.127**	**0.002**
Digital information seeking frequency score	−0.044	0.342	−0.056	0.238	−0.037	0.431
COVID‐19 negative sentiments score	0.005	0.887	−0.009	0.789	0.007	0.831
COVID‐19 negative sentiments score						
Traditional information seeking frequency score	−0.007	0.861	−0.018	0.679	−0.020	0.630
Digital information seeking frequency score	−0.089	0.051	−0.084	0.072	−0.071	0.123
Number of observations	925		871		925	
Number of parameters	115		113		112	
Log likelihood	−34757.323		−32839.807		−37165.594	
RMSEA	0.047		0.057		0.046	
	[0.044, 0.050]		[0.054, 0.060]		[0.043, 0.049]	
SRMR	0.043		0.045		0.040	
CFI	0.941		0.922		0.952	
TLI	0.931		0.909		0.944	
$\chi^{2}$	1195.597		1487.927		1169.449	
p‐Value	< 0.001		< 0.001		< 0.001	

*Note:* Bold values represent significant results at *p* < 0.05.

While the frequency of information seeking on traditional media had a positive effect on conspiracy beliefs (*p* = 0.003, *p* = 0.002, and *p* < 0.001, respectively), information seeking on digital media and negative sentiments had no significant effect on this variable^2^. Therefore, an inverse effect between traditional information seeking and conspiracy beliefs was found (H5b not confirmed) and no relationships between digital information seeking and conspiracy beliefs (H5a not confirmed) and negative sentiments and conspiracy beliefs (H8 not confirmed) were found. Institutional mistrust was positively influenced by negative sentiments (*p* < 0.001, *p* < 0.001, and *p* < 0.001, respectively, then confirming H9) but unrelated to information seeking on traditional and digital media (not confirming H6a and H6b). Finally, neither traditional nor digital information seeking influenced negative sentiments, providing no support H7a and H7b.

Gender, age, school attendance, cohabitation with a partner, employment status (paid employment or self‐employed), and urban residency were significant predictors depending on the specific analysis. In terms of socio‐economic and demographic variables, several factors were found to be associated with containment‐related behaviours. Specifically, being female, attending school, living with a partner, and having a salaried employment had a positive effect on the adoption of containment‐related behaviours (*p* = 0.012, *p* = 0.042, *p* < 0.001, *p* < 0.001, respectively). On the other hand, residing in an urban area appeared to decrease the likelihood of engaging in these behaviours (*p* = 0.028). Additionally, having a salaried employment was found to influence agreement with sanitary measures (*p* = 0.002), while individuals living in urban areas displayed less favourable attitudes towards vaccination (*p* = 0.044). Furthermore, these socio‐economic and demographic variables had a significant impact on institutional mistrust. Specifically, individuals who went to school, lived in urban areas, and held salaried employment exhibited higher levels of mistrust towards institutions. All results as well as other models' specifications are presented in Online Supporting Information S1: File 5.

## 5 Discussion

The first objective of this paper was to study how trust and conspiracy beliefs directly influence COVID‐19 preventive behaviours. Our findings underscore the significant adverse impact of institutional mistrust and endorsement of conspiracy beliefs on containment‐related behaviours, attitudes towards vaccination, and agreement with sanitary measures among the population of Burkina Faso. Several studies previously underlined similar results for containment‐related behaviours [22, 89] or vaccination in high‐ and middle‐income countries [28, 42]. Our study, like theirs in other contexts, highlight the crucial importance of addressing these factors in public health interventions and communication strategies in Burkina Faso. To the best of our knowledge, our research is the first in Sub‐Saharan countries to demonstrate that institutional trust has both direct and indirect effects on COVID‐19 health‐related behaviours. This underscores the need for medium‐to long‐term policies aimed at restoring and perpetuating trust in institutions and curbing conspiracy beliefs in the low‐compliance population. In the meantime, prevention and promotion campaigns aiming at fostering the adoption of protective health behaviours should be channelled through the sources of information in which individuals have the greatest confidence, specifically religious and traditional leaders that benefited from the higher level of trust in our survey (Figure 2, Online Supporting Information S1: File 4). Kohler et al. (2022) [90] emphasised the pivotal role of community leadership in fostering better compliance and adoption of public health essential measures to contain the virus in Malawi, which is also a fairly general conclusion to be drawn from the literature on factors favouring vaccine acceptance in LMICs [91]. The positive role of traditional and religious leaders was also very influential during the fight against Ebola [92, 93]. It is therefore appropriate that the Burkina Faso government should seek to rely more on traditional and religious leaders as mediators in its communication strategy, but as long as they support vaccination, which is not the case for all of them in Burkina Faso. The literature also points to the need to adapt communication to the specificities of target groups and design culturally targeted interventions [94, 95]. This is an acute issue in Burkina Faso, where there are significant differences between population groups (including ethnic groups) with various characteristics and corpus of social norms that contribute to shaping their perception of the virus and their adherence to containment measures [96].

Furthermore, we found that the direct effect of mistrust on health‐related outcomes was stronger than the effect of conspiracy beliefs except in the case of vaccination attitudes (see Figure 4, Online Supporting Information S1: Files 3 and 5). Special attention must be given to the COVID‐19 pandemic, as well as health crises in general, to prevent them from becoming breeding grounds for conspiracy beliefs that could hinder vaccination campaigns. This is particularly relevant in the context of African countries where childhood vaccination is already suboptimal with 1 in 5 children not receiving all the necessary vaccines leading to the death of 500,000 million of children each year from vaccine‐preventable diseases. In this context, studies should be conducted in Sub‐Saharan Africa to explore whether parental vaccination attitudes towards childhood vaccinations evolved since the beginning of the COVID‐19 pandemic, and to investigate the drivers of this potential change in attitudes. Indeed, increased defiance towards childhood vaccinations could fuel the spread of infectious diseases among children and raise a new threat on public health in African countries at a time the COVID‐19 pandemic is not yet completely under control. Our findings also align with existing literature by demonstrating a positive association between institutional mistrust and conspiratorial beliefs. By establishing this relationship, our study contributes to the limited body of research in LMICs, thereby enriching the current knowledge in this area. This positive association between mistrust and conspiratorial beliefs increases the complexity of tackling them. A review of the effectiveness of interventions to reduce beliefs in conspiracy theories [97] showed that rational counterarguments that describe the factual inaccuracies of conspiracy theories have only a small or very small effect on beliefs, and that most existing interventions are ineffective. Their results suggested that the most effective anti‐conspiracy interventions were those that took place before people had been exposed to conspiracy beliefs, calling for an emphasis on informational inoculation consisting of a pre‐emptive debunking. This is a major challenge for Burkina Faso, where the adult literacy rate and the gross secondary school enrolment rate are particularly low, at 46% and 39% respectively by 2021 (World Bank Data Indicators). In such a context, Bono et al.'s [9] suggestion to target low‐income households through community approaches by health authorities to dispel misinformation regarding COVID‐19 vaccines sounds very relevant for Burkina Faso.

Our second objective was to explore the antecedents of trust and conspiracy beliefs to build a comprehensive model of COVID‐19 preventive behaviours adoption. Most of the hypotheses formulated based on the existing literature, primarily from high‐income countries, were not supported. This may indicate that the context in Burkina Faso, as well as other LMIC contexts, presents specific characteristics that influence the relationships between health‐related behaviours, mistrust, and conspiracy beliefs. First, contrary to what was found in high‐income countries [64], China [65, 66, 67, 68] or even Ghana [69], our results did not provide evidence on the relationship between information seeking and institutional mistrust in Burkina Faso. This might be partly linked to the measures used in our study. Indeed, our traditional media measure aggregated all types of radio, TV channels and newspapers while previous literature found that the type of ownership (public vs. commercial) mattered for political trust. The digital media measure we used also gathered social media and general search engines while Ceron (2015) [71] found that websites and social media had opposite relationships with political trust. When running our analyses using social media and general search engine alternately instead of the aggregated measure of digital media, we find a negative and significant association between those social media use and institutional trust (results available from the authors upon request). A second important finding pertains to the relationship between information‐seeking behaviour and conspiracy beliefs. While traditional media consumption exhibited a positive association with conspiracy beliefs, digital media consumption did not show a significant association. This is in contrast with previous literature conducted in high‐income countries which found a positive and negative effect of traditional and social media on conspiracy beliefs, respectively [59, 60, 61, 62, 63]. This nuanced understanding of the influence of different information sources contributes to the literature on the role of media and information dissemination during a pandemic. However, this finding could be attributed to various factors, such as limited use of social networks for information seeking in low‐income setting such as Burkina Faso (where internet users accounted for only 22% of the population in 2021 according to World Bank data) or measurement limitations for digital information. Furthermore, while our study did not evidence a link between negative sentiments and conspiracy beliefs, we found that negative emotions had a positive effect on institutional mistrust as was previously documented in the US [79, 98]. In summary, the hypotheses we formulated regarding the relationship between information seeking, institutional mistrust, and conspiracy beliefs were not supported by our findings, for two main reasons that cannot be clearly disentangled. First, these hypotheses were derived from a body of literature predominantly based in high‐income countries, where patterns of traditional and digital media use differ substantially from those in our context. Second, our measures aggregated multiple types of media, whereas prior studies often conducted media‐specific analyses, potentially capturing more nuanced effects. However, our results underscore the significance of implementing supportive economic and psychological policies for the population in the time of pandemics to mitigate the rise of institutional mistrust and improve adherence to official recommendations aimed at controlling the pandemic.

Regarding specific demographics, individuals with salaried employment were more likely to adopt containment‐related behaviours. Compliance with hygiene practices appears less costly in terms of perceived benefits to individuals compared to pharmaceutical interventions or policies, which may explain the preference for such behaviours. Additionally, adherence to hygiene rules may be fostered more effectively within the formal sector compared to the informal sector. Individuals with higher levels of education and salaried employment were more likely to express institutional mistrust. This suggests that better socioeconomic standing may contribute to institutional mistrust. We did not find any significant effect of socioeconomic background on conspiracy beliefs.

Our study has several strengths that contribute to a comprehensive analysis of COVID‐19 health attitudes and behaviours in Burkina Faso. We took a multi‐dimensional approach, considering various factors that could potentially influence individuals' responses to the pandemic. This approach adds depth and richness to our analysis, providing a more nuanced understanding of the relationships and mechanisms at play. By exploring multiple dimensions, we captured a more comprehensive picture of the influences on containment‐related behaviours, agreement with sanitary measures, vaccination attitudes, and other outcomes of interest. However, our study also has some limitations that should be acknowledged. First, the findings are based on a subset of individuals who chose to participate and that is not fully representative of the Burkinabe population, potentially affecting the generalisability of our results (Table 1). Indeed, our sample was skewed towards males and urban residents. This may be due to cultural and religious issues and insecurity (local armed conflicts) in several areas in Sahel, East, and North regions at the time of the survey. However, subgroup analyses by gender and living area did not reveal differences between those subgroups. Besides, face to face interviews through which data were collected might have generated a desirability bias in some of respondents' responses. However, the use of self‐administered questionnaires (either paper or online questionnaires) to conduct the survey, which would have reduced the desirability bias, was not possible for this study given that a significant share of the Burkinabe population is not literate and that access to the internet is low in rural areas. Furthermore, our study utilised a cross‐sectional research design, which captures data at a specific point in time. This design limitation hinders our ability to establish causal relationships between the variables of interest. Longitudinal studies would be valuable for examining changes in attitudes and behaviours over time and are needed to investigate health‐related behaviours related to conspiracy beliefs and trust in African countries. Lastly, it is important to note that our study may not account for all potential confounding variables that could influence the relationships between the variables such as political adherence or trust in information. There may be omitted or unmeasured factors that could impact the observed associations and limit the accuracy of our findings. Despite these limitations, our study offers valuable insights into the complex interplay of various factors influencing COVID‐19 attitudes and behaviours and provides useful insights for African policy makers regarding the design of prevention and information campaigns.

## Author Contributions

Conceptualisation: M.G., J.M. Methodology: G.M., M.G. Software: G.M., M.G. Validation: G.M., M.G. Formal analysis: G.M. Investigation: G.M., M.G. Resources: J.M., M.G. Data Curation: G.M., M.G. Writing – Original Draft: G.M., M.G. Writing – Review and Editing: J.M., M.G., G.M. Visualisation: G.M. Supervision: M.G., J.M. Project administration: J.M. Funding acquisition: J.M.

## Funding

This study was financed by the FERDI, the CERDI and the Agence Nationale de la Recherche (France) as part of the programme “Investments for the Future'' ANR‐10‐LABX‐14‐01.

## Conflicts of Interest

The authors declare no conflicts of interest.

## Supporting information


Supporting Information S1

## Data Availability

The data that support the findings of this study are available from the corresponding author upon reasonable request.

## Endnotes

^1^
International Monetary Fund.

^2^
When considering social networks and websites separately instead of the aggregated measure, a significant negative effect of social networks on negative sentiments was found (at *p* < 0.05) and a weaker negative significant (at *p* < 0.10) effect of the use of websites on conspiracy beliefs was found.
