# Supplementary material for: Conspiracy Beliefs, Institutional Mistrust, and Health‐Related Behaviours During the COVID‐19 Pandemic in Burkina Faso: A Mediation Analysis
Source: Int J Health Plann Manage. 2026 Jan 28;41(3):461–76. doi: 10.1002/hpm.70058 (PMC13127567; doi:10.1002/hpm.70058)
Supplement: Supplementary file 1 — Supporting Information S1 [file HPM-41-461-s001.docx]

**Title: Conspiracy beliefs, institutional mistrust, and health-related behaviors during the COVID-19 pandemic in Burkina Faso: a mediation analysis.**

**Supplementary files**

[Supplementary File 1 – Hypothesis references 2](#_Toc219452401)

[Supplementary File 2 – Bivariate analysis between latent variables (scored) and socio-economic and demographic variables 5](#_Toc219452402)

[Supplementary File 3 – Direct, indirect, and total effects of the mediation analyses 7](#_Toc219452403)

[Supplementary File 4 – Factor analysis of latent variables considered in the Structural Equations Models 8](#_Toc219452404)

[Supplementary File 5 – Complete structural equations models with specifications 10](#_Toc219452405)

# **Supplementary File 1 – Hypothesis references**

22. Algan Y, Cohen D, Davoine E, Foucault M, Stantcheva S. Trust in scientists in times of pandemic: Panel evidence from 12 countries. Proc Natl Acad Sci U S A. 2021;118(40):e2108576118. doi:10.1073/pnas.2108576118

23. Latkin CA, Dayton L, Yi G, Konstantopoulos A, Boodram B. Trust in a COVID-19 vaccine in the U.S.: A social-ecological perspective. Soc Sci Med 1982. 2021;270:113684. doi:10.1016/j.socscimed.2021.113684

24. Trent M, Seale H, Chughtai AA, Salmon D, MacIntyre CR. Trust in government, intention to vaccinate and COVID-19 vaccine hesitancy: A comparative survey of five large cities in the United States, United Kingdom, and Australia. Vaccine. 2022;40(17):2498-2505. doi:10.1016/j.vaccine.2021.06.048

25. Wu J, Li Q, Silver Tarimo C, et al. COVID-19 Vaccine Hesitancy Among Chinese Population: A Large-Scale National Study. Front Immunol. 2021;12:781161. doi:10.3389/fimmu.2021.781161

26. Ye Y, Su Z, Shi C. Institutional trust, scientific literacy, and information sources: What factors determine people’s attitudes toward COVID-19 vaccines of different origins in China? Front Public Health. 2023;11:1092425. doi:10.3389/fpubh.2023.1092425

27. Nomura S, Eguchi A, Yoneoka D, et al. Reasons for being unsure or unwilling regarding intention to take COVID-19 vaccine among Japanese people: A large cross-sectional national survey. Lancet Reg Health West Pac. 2021;14:100223. doi:10.1016/j.lanwpc.2021.100223

28. Van Oost P, Yzerbyt V, Schmitz M, et al. The relation between conspiracism, government trust, and COVID-19 vaccination intentions: The key role of motivation. Soc Sci Med 1982. 2022;301:114926. doi:10.1016/j.socscimed.2022.114926

29. Guillon M, Kergall P. Factors associated with COVID-19 vaccination intentions and attitudes in France. Public Health. 2021;198:200-207. doi:10.1016/j.puhe.2021.07.035

30. Seddig D, Maskileyson D, Davidov E, Ajzen I, Schmidt P. Correlates of COVID-19 vaccination intentions: Attitudes, institutional trust, fear, conspiracy beliefs, and vaccine skepticism. Soc Sci Med 1982. 2022;302:114981. doi:10.1016/j.socscimed.2022.114981

31. Murphy J, Vallières F, Bentall RP, et al. Psychological characteristics associated with COVID-19 vaccine hesitancy and resistance in Ireland and the United Kingdom. Nat Commun. 2021;12(1):29. doi:10.1038/s41467-020-20226-9

32. Rodriguez-Morales AJ, Franco OH. Public trust, misinformation and COVID-19 vaccination willingness in Latin America and the Caribbean: today’s key challenges. Lancet Reg Health Am. 2021;3:100073. doi:10.1016/j.lana.2021.100073

33. Morales-García WC, Huancahuire-Vega S, Saintila J, Morales-García M, Fernández-Molocho L, Ruiz Mamani PG. Predictors of Intention to Vaccinate Against COVID-19 in a Peruvian Sample. J Prim Care Community Health. 2022;13:21501319221092254. doi:10.1177/21501319221092254

34. Karabela ŞN, Coşkun F, Hoşgör H. Investigation of the relationships between perceived causes of COVID-19, attitudes towards vaccine and level of trust in information sources from the perspective of Infodemic: the case of Turkey. BMC Public Health. 2021;21(1):1195. doi:10.1186/s12889-021-11262-1

35. Ghaddar A, Khandaqji S, Awad Z, Kansoun R. Conspiracy beliefs and vaccination intent for COVID-19 in an infodemic. PloS One. 2022;17(1):e0261559. doi:10.1371/journal.pone.0261559

36. Cooper S, van Rooyen H, Wiysonge CS. COVID-19 vaccine hesitancy in South Africa: how can we maximize uptake of COVID-19 vaccines? Expert Rev Vaccines. 2021;20(8):921-933. doi:10.1080/14760584.2021.1949291

37. Tobin EA, Okonofua M, Adeke A, Obi A. Willingness to Accept a COVID-19 Vaccine in Nigeria: A Population-based Cross-sectional Study. Cent Afr J Public Health. 2021;7(2):53. doi:10.11648/j.cajph.20210702.12

38. Alhassan RK, Aberese-Ako M, Doegah PT, et al. COVID-19 vaccine hesitancy among the adult population in Ghana: evidence from a pre-vaccination rollout survey. Trop Med Health. 2021;49(1):96. doi:10.1186/s41182-021-00357-5

39. Wang Q, Yang L, Jin H, Lin L. Vaccination against COVID-19: A systematic review and meta-analysis of acceptability and its predictors. Prev Med. 2021;150:106694. doi:10.1016/j.ypmed.2021.106694

40. Loomba S, de Figueiredo A, Piatek SJ, de Graaf K, Larson HJ. Measuring the impact of COVID-19 vaccine misinformation on vaccination intent in the UK and USA. Nat Hum Behav. 2021;5(3):337-348. doi:10.1038/s41562-021-01056-1

41. Ruiz JB, Bell RA. Predictors of intention to vaccinate against COVID-19: Results of a nationwide survey. Vaccine. 2021;39(7):1080-1086. doi:10.1016/j.vaccine.2021.01.010

42. Freeman D, Loe BS, Chadwick A, et al. COVID-19 vaccine hesitancy in the UK: the Oxford coronavirus explanations, attitudes, and narratives survey (Oceans) II. Psychol Med. 2022;52(14):3127-3141. doi:10.1017/S0033291720005188

43. Yang Z, Luo X, Jia H. Is It All a Conspiracy? Conspiracy Theories and People’s Attitude to COVID-19 Vaccination. Vaccines. 2021;9(10):1051. doi:10.3390/vaccines9101051

44. Gao J, Raza SH, Yousaf M, Shah AA, Hussain I, Malik A. How Does Digital Media Search for COVID-19 Influence Vaccine Hesitancy? Exploring the Trade-off between Google Trends, Infodemics, Conspiracy Beliefs and Religious Fatalism. Vaccines. 2023;11(1):114. doi:10.3390/vaccines11010114

45. Jin Q, Raza SH, Yousaf M, Zaman U, Siang JMLD. Can Communication Strategies Combat COVID-19 Vaccine Hesitancy with Trade-Off between Public Service Messages and Public Skepticism? Experimental Evidence from Pakistan. Vaccines. 2021;9(7):757. doi:10.3390/vaccines9070757

46. El-Elimat T, AbuAlSamen MM, Almomani BA, Al-Sawalha NA, Alali FQ. Acceptance and attitudes toward COVID-19 vaccines: A cross-sectional study from Jordan. PloS One. 2021;16(4):e0250555. doi:10.1371/journal.pone.0250555

47. Kaspar K, Nordmeyer L. Personality and Motivation to Comply With COVID-19 Protective Measures in Germany. Front Psychol. 2022;13. Accessed March 10, 2023. https://www.frontiersin.org/articles/10.3389/fpsyg.2022.893881

48. Allington D, Duffy B, Wessely S, Dhavan N, Rubin J. Health-protective behaviour, social media usage and conspiracy belief during the COVID-19 public health emergency. Psychol Med. 2021;51(10):1763-1769. doi:10.1017/S003329172000224X

49. Gerace A, Rigney G, Anderson JR. Predicting attitudes towards easing COVID-19 restrictions in the United States of America: The role of health concerns, demographic, political, and individual difference factors. PloS One. 2022;17(2):e0263128. doi:10.1371/journal.pone.0263128

50. Abalakina-Paap M, Stephan WG, Craig T, Gregory WL. Beliefs in Conspiracies. Polit Psychol. 1999;20(3):637-647. doi:10.1111/0162-895X.00160

51. Imhoff R, Lamberty P. How paranoid are conspiracy believers? Toward a more fine-grained understanding of the connect and disconnect between paranoia and belief in conspiracy theories. Eur J Soc Psychol. 2018;48(7):909-926. doi:10.1002/ejsp.2494

52. Goertzel T. Belief in Conspiracy Theories. Polit Psychol. 1994;15(4):731-742. doi:10.2307/3791630

53. Bruder M, Kunert L. The conspiracy hoax? Testing key hypotheses about the correlates of generic beliefs in conspiracy theories during the COVID-19 pandemic. Int J Psychol J Int Psychol. 2022;57(1):43-48. doi:10.1002/ijop.12769

54. Pummerer L, Böhm R, Lilleholt L, Winter K, Zettler I, Sassenberg K. Conspiracy Theories and Their Societal Effects During the COVID-19 Pandemic. Soc Psychol Personal Sci. 2022;13(1):49-59. doi:10.1177/19485506211000217

55. Kim S, Kim S. Searching for General Model of Conspiracy Theories and Its Implication for Public Health Policy: Analysis of the Impacts of Political, Psychological, Structural Factors on Conspiracy Beliefs about the COVID-19 Pandemic. Int J Environ Res Public Health. 2021;18(1):266. doi:10.3390/ijerph18010266

56. Martinez AP, Shevlin M, Valiente C, Hyland P, Bentall RP. Paranoid beliefs and conspiracy mentality are associated with different forms of mistrust: A three-nation study. Front Psychol. 2022;13. Accessed April 4, 2023. https://www.frontiersin.org/articles/10.3389/fpsyg.2022.1023366

57. Vosoughi S, Roy D, Aral S. The spread of true and false news online. Science. 2018;359(6380):1146-1151. doi:10.1126/science.aap9559

58. Douglas KM, Uscinski JE, Sutton RM, et al. Understanding Conspiracy Theories. Polit Psychol. 2019;40(S1):3-35. doi:10.1111/pops.12568

59. De Coninck D, Frissen T, Matthijs K, et al. Beliefs in Conspiracy Theories and Misinformation About COVID-19: Comparative Perspectives on the Role of Anxiety, Depression and Exposure to and Trust in Information Sources. Front Psychol. 2021;12. Accessed April 12, 2023. https://www.frontiersin.org/articles/10.3389/fpsyg.2021.646394

60. Romer D, Jamieson KH. Patterns of Media Use, Strength of Belief in COVID-19 Conspiracy Theories, and the Prevention of COVID-19 From March to July 2020 in the United States: Survey Study. J Med Internet Res. 2021;23(4):e25215. doi:10.2196/25215

61. Romer D, Jamieson KH. Conspiracy theories as barriers to controlling the spread of COVID-19 in the U.S. Soc Sci Med 1982. 2020;263:113356. doi:10.1016/j.socscimed.2020.113356

62. Stecula DA, Pickup M. Social Media, Cognitive Reflection, and Conspiracy Beliefs. Front Polit Sci. 2021;3. Accessed April 12, 2023. https://www.frontiersin.org/articles/10.3389/fpos.2021.647957

63. Strömbäck J, Broda E, Bouchafra S, Johansson S, Rettenegger G, Lindgren E. Conspiracy thinking and the role of media use: Exploring the antecedents of conspiratorial predispositions. Eur J Commun. Published online August 29, 2022:02673231221122951. doi:10.1177/02673231221122951

64. Verboord M. Bundles of trust? Examining the relationships between media repertoires, institutional trust, and social contexts. Communications. Published online March 1, 2023. doi:10.1515/commun-2022-0013

65. Xu P, Ye Y, Zhang M. Exploring the effects of traditional media, social media, and foreign media on hierarchical levels of political trust in China. Glob Media China. 2022;7(3):357-377. doi:10.1177/20594364221115270

66. Meng X, Li Y. Parsing the Relationship Between Political News Consumption and Hierarchical Political Trust in China. Journal Pract. 2022;16(7):1363-1382. doi:10.1080/17512786.2020.1867623

67. Zhou D, Deng W, Wu X. Impacts of Internet Use on Political Trust: New Evidence from China. Emerg Mark Finance Trade. 2020;56(14):3235-3251. doi:10.1080/1540496X.2019.1644161

68. Huang Y, Wang L. Political Values and Political Trust in the Digital Era: How Media Engagement Divides Chinese Netizens. Int J Sociol. 2021;51(3):197-217. doi:10.1080/00207659.2021.1881870

69. Wahab GA. Partisanship, News Use, and Political Attitudes in Ghana: An Application of the Communication Mediation Model. Afr Journal Stud. 2021;42(3):113-130. doi:10.1080/23743670.2021.1884580

70. Strömbäck J, Djerf-Pierre M, Shehata A. A Question of Time? A Longitudinal Analysis of the Relationship between News Media Consumption and Political Trust. Int J Press. 2016;21(1):88-110. doi:10.1177/1940161215613059

71. Ceron A. Internet, News, and Political Trust: The Difference between Social Media and Online Media Outlets. J Comput-Mediat Commun. 2015;20(5):487-503. doi:10.1111/jcc4.12129

72. Shensa A, Sidani JE, Dew MA, Escobar-Viera CG, Primack BA. Social Media Use and Depression and Anxiety Symptoms: A Cluster Analysis. Am J Health Behav. 2018;42(2):116-128. doi:10.5993/AJHB.42.2.11

73. Hwang J, Borah P, Shah D, Brauer M. The Relationship among COVID-19 Information Seeking, News Media Use, and Emotional Distress at the Onset of the Pandemic. Int J Environ Res Public Health. 2021;18(24):13198. doi:10.3390/ijerph182413198

74. Akca A, Ayaz-Alkaya S. Media use, positive and negative emotions, and stress levels of adults during COVID-19 pandemic in Turkey: A cross-sectional study. Int J Nurs Pract. 2022;28(2):e13035. doi:10.1111/ijn.13035

75. Kim K, Yang J, Jeon YJ, et al. The effects of information-seeking behaviours on prevention behaviours during the COVID-19 pandemic: the mediating effects of anxiety and fear in Korea. Epidemiol Health. 2021;43:e2021085. doi:10.4178/epih.e2021085

76. van Prooijen J, Douglas KM. Belief in conspiracy theories: Basic principles of an emerging research domain. Eur J Soc Psychol. 2018;48(7):897-908. doi:10.1002/ejsp.2530

77. Molenda Z, Green R, Marchlewska M, Cichocka A, Douglas KM. Emotion dysregulation and belief in conspiracy theories. Personal Individ Differ. 2023;204:112042. doi:10.1016/j.paid.2022.112042

78. Dunn JR, Schweitzer ME. Feeling and believing: the influence of emotion on trust. J Pers Soc Psychol. 2005;88(5):736-748. doi:10.1037/0022-3514.88.5.736

79. Myers CD, Tingley D. The Influence of Emotion on Trust. Polit Anal. 2017;24(4):492-500. doi:10.1093/pan/mpw026

# **Supplementary File 2 – Bivariate analysis between latent variables (scored) and socio-economic and demographic variables**

|  | **Vaccination Attitudes score**^2^ | | | | | **Containment Related Behaviors score**^3^ | | | | | **Agreement with Sanitary Measures score**^4^ | | | | |
| --- | --- | --- | --- | --- | --- | --- | --- | --- | --- | --- | --- | --- | --- | --- | --- |
|  | **N** | **Mean** | **SD** | **Pearson** $\boldsymbol{r}$ | $\boldsymbol{p}$**-value^1^** | **N** | **Mean** | **SD** | **Pearson** $\boldsymbol{r}$ | $\boldsymbol{p}$**-value^1^** | **N** | **Mean** | **SD** | **Pearson** $\boldsymbol{r}$ | $\boldsymbol{p}$**-value^1^** |
| Gender |  |  |  |  |  |  |  |  |  |  |  |  |  |  |  |
| Male | 627 | 22.188 | 9.961 |  | **<.001** | 585 | 7.545 | 5.564 |  | **0.038** | 591 | 7.775 | 6.260 |  | **<.001** |
| Female | 347 | 24.686 | 9.584 |  |  | 330 | 8.327 | 5.292 |  |  | 326 | 9.353 | 6.023 |  |  |
| Age (in years) | 974 |  |  | 0.132 | **<.001** | 915 |  |  | 0.066 | **0.046** | 917 |  |  | 0.087 | **0.008** |
| Attended school |  |  |  |  |  |  |  |  |  |  |  |  |  |  |  |
| Yes | 617 | 21.535 | 9.304 |  | **<.001** | 581 | 7.950 | 5.374 |  | 0.379 | 593 | 7.703 | 6.092 |  | **<.001** |
| No | 356 | 25.705 | 10.312 |  |  | 333 | 7.619 | 5.663 |  |  | 323 | 9.477 | 6.294 |  |  |
| Marital status |  |  |  |  |  |  |  |  |  |  |  |  |  |  |  |
| Live alone | 701 | 24.144 | 9.889 |  | **<.001** | 658 | 7.848 | 5.527 |  | 0.855 | 646 | 8.825 | 6.229 |  | **<.001** |
| Live with a partner | 273 | 20.341 | 9.392 |  |  | 257 | 7.774 | 5.359 |  |  | 271 | 7.170 | 6.050 |  |  |
| Salaried employment |  |  |  |  |  |  |  |  |  |  |  |  |  |  |  |
| Yes | 236 | 21.309 | 9.448 |  | **0.002** | 227 | 9.167 | 5.212 |  | **<.001** | 225 | 8.578 | 6.250 |  | 0.502 |
| No | 738 | 23.644 | 9.975 |  |  | 688 | 7.385 | 5.495 |  |  | 692 | 8.257 | 6.212 |  |  |
| Self-Employed |  |  |  |  |  |  |  |  |  |  |  |  |  |  |  |
| Yes | 288 | 22.139 | 9.641 |  | 0.063 | 268 | 7.847 | 5.709 |  | 0.952 | 267 | 7.213 | 6.144 |  | **0.001** |
| No | 683 | 23.432 | 9.969 |  |  | 644 | 7.823 | 5.395 |  |  | 648 | 8.775 | 6.193 |  |  |
| Type of residency |  |  |  |  |  |  |  |  |  |  |  |  |  |  |  |
| Rural | 585 | 24.805 | 9.868 |  | **<.001** | 554 | 8.249 | 5.670 |  | **0.009** | 554 | 8.966 | 6.212 |  | **<.001** |
| Urban | 387 | 20.452 | 9.381 |  |  | 359 | 7.142 | 5.098 |  |  | 361 | 7.388 | 6.129 |  |  |
| Conspiracy Beliefs^5^ | 972 |  |  | -0.494 | **<.001** | 913 |  |  | -0.224 | **<.001** | 916 |  |  | -0.248 | **<.001** |
| Institutional Mistrust^6^ | 968 |  |  | -0.378 | **<.001** | 910 |  |  | -0.229 | **<.001** | 911 |  |  | -0.333 | **<.001** |
| Traditional Information Seeking^7^ | 961 |  |  | -0.151 | **<.001** | 905 |  |  | 0.036 | 0.287 | 906 |  |  | -0.115 | **0.001** |
| Digital Information Seeking^8^ | 967 |  |  | -0.194 | **<.001** | 909 |  |  | 0.029 | 0.381 | 911 |  |  | -0.087 | **0.009** |
| COVID-19 Negative Sentiments^9^ | 952 |  |  | -0.055 | 0.093 | 892 |  |  | 0.028 | 0.411 | 895 |  |  | -0.117 | **<.001** |

^1^One-way analysis of variance and Kruskal-Wallis H test performed according to the equality of variances between categorical and continuous variables. Student $t$ test performed between continuous variables.

^2^COVID-19 Vaccination Attitudes were measured using an 8-item questionnaire, each item ranging from 1 to 5. For bivariate analyses, a score ranging from 8 (negative attitudes toward COVID-19 vaccination) to 40 (positive attitudes toward COVID-19 vaccination) was computed by summing each item. Cronbach’s α was equal to $.9563$.

^3^Containment-related Behaviors were assessed using an 8-item questionnaire, each item ranging from 0 to 2. For bivariate analyses, a score ranging from 0 (low application of containment-related behaviors) to 16 (high application of containment-related behaviors) was computed by summing each item. Cronbach’s α was equal to $.9307$.

^4^Agreement with Sanitary Measures was assessed using an 8-item questionnaire, each item ranging from 0 to 2. For bivariate analyses, a score ranging from 0 (low agreement) to 16 (high agreement) was computed by summing each item. Cronbach’s α was equal to $.9409$.

^5^COVID-19 Conspiracy Beliefs were measured using a 7-item questionnaire, each item ranging from 1 to 5. For bivariate analyses, a score ranging from 7 (low conspiracy beliefs) to 35 (high conspiracy beliefs) was computed by summing each item. Cronbach’s α was equal to $.9186$.

^6^Institutional Mistrust was assessed using a 7-item questionnaire, each item ranging from 1 to 5. For bivariate analyses, a score ranging from 7 (low mistrust) to 35 (high mistrust) was computed by summing each item. Cronbach’s α was equal to $.8406$.

^7^Traditional information seeking was computed by summing the frequency (from 0 “Never” to 4 “Every day”) of radio, television, and print press use. The score ranges from 0 to 12.

^8^Digital information seeking was computed by summing the frequency (from 0 “Never” to 4 “Every day”) of social networks and websites use. The score ranges from 0 to 8.

^9^COVID-19 negative sentiments were assessed by summing four items scaled between 0 and 1: satisfaction with life, fear, hope, and anger. The score ranges from 0 to 4 and Cronbach’s α was equal to $.3596$.

# **Supplementary File 3 – Direct, indirect, and total effects of the mediation analyses**

The Z coefficient corresponds to the direct effect.

The product of $X\times Y$ corresponds to the indirect effect.

The total effect is given by the sum of the direct and indirect effects.

|  | **Containment-related Behaviors** | **Agreement with Sanitary Measures** | **COVID-19 Vaccine Attitudes** |
| --- | --- | --- | --- |
| Direct effect | -0.2059 | -0.2939 | -0.2275 |
| Indirect effect | -0.0456 | -0.0392 | -0.1169 |
| Total effect | -0.2514 | -0.3331 | -0.3444 |
| Ratio of the indirect effect to the total effect | 0.1812 | 0.1176 | 0.3393 |
| Ratio of the indirect effect to the direct effect | 0.2213 | 0.1332 | 0.5136 |

# **Supplementary File 4 – Factor analysis of latent variables considered in the Structural Equations Models**

| **Latent variables** | **Bartlett test of sphericity (**$\boldsymbol{p}$**-value)** | **KMO Measure of Sampling Adequacy** |
| --- | --- | --- |
| 1. COVID-19 Conspiracy Beliefs | <.001 | 0.868 |
| 2. Institutional Mistrust | <.001 | 0.769 |
| 3. Containment-Related Behaviors (CRB) | <.001 | 0.909 |
| 4. COVID-19 Agreement with Sanitary Measures | <.001 | 0.922 |
| 5. COVID-19 Vaccination Attitudes | <.001 | 0.956 |

| **Containment-related Behaviors (CRB)** | **Mean** | **Standard deviation** | $\boldsymbol{p}$**-value (**$\boldsymbol{H}_{\boldsymbol{0}}\boldsymbol{:}\overline{\boldsymbol{x}}\boldsymbol{=1}$**)** | **Median** | **Range** | **Factor 1** | **Factor 2** | **Factor 3** | **Factor 4** | **Uniqueness** | **1.** | **2.** | **3.** | **4.** | **5.** | **6.** | **7.** | **8.** | **Item-test correlation** | **Item-rest correlation** | **Inter-item correlation** | **Cronbach's** $\boldsymbol{\alpha}$ **if item removed** |
| --- | --- | --- | --- | --- | --- | --- | --- | --- | --- | --- | --- | --- | --- | --- | --- | --- | --- | --- | --- | --- | --- | --- |
| CRB1. You wash your hands more often and/or for longer | 1.403 | 0.711 | <.0001 | 2 | (0-2) | 0.2751 | 0.7198 | 0.1177 | 0.0460 | 0.3903 | 1.000 |  |  |  |  |  |  |  | 0.6874 | 0.6052 | 0.4654 | 0.9318 |
|  |  |  |  |  |  |  |  |  |  |  | - |  |  |  |  |  |  |  |  |  |  |  |
| CRB2. Coughing or sneezing into your elbow or a handkerchief | 1.209 | 0.785 | <.0001 | 1 | (0-2) | 0.3227 | 0.7328 | 0.1492 | 0.0265 | 0.3358 | 0.690 | 1.000 |  |  |  |  |  |  | 0.7412 | 0.6625 | 0.4466 | 0.9284 |
|  |  |  |  |  |  |  |  |  |  |  | <.001 | - |  |  |  |  |  |  |  |  |  |  |
| CRB3. You have stopped shaking hands in greeting | 1.003 | 0.810 | 0.9059 | 1 | (0-2) | 0.5572 | 0.4661 | 0.4045 | 0.0596 | 0.3050 | 0.521 | 0.578 | 1.000 |  |  |  |  |  | 0.8331 | 0.7757 | 0.4241 | 0.9200 |
|  |  |  |  |  |  |  |  |  |  |  | <.001 | <.001 | - |  |  |  |  |  |  |  |  |  |
| CRB4. You keep a distance of one meter from other people | 0.873 | 0.817 | <.0001 | 1 | (0-2) | 0.5746 | 0.4556 | 0.4027 | 0.0322 | 0.2991 | 0.503 | 0.578 | 0.735 | 1.000 |  |  |  |  | 0.8371 | 0.7808 | 0.4223 | 0.9197 |
|  |  |  |  |  |  |  |  |  |  |  | <.001 | <.001 | <.001 | - |  |  |  |  |  |  |  |  |
| CRB5. You have reduced your travels | 0.914 | 0.884 | 0.0024 | 1 | (0-2) | 0.7342 | 0.3949 | 0.2174 | 0.2379 | 0.2012 | 0.506 | 0.539 | 0.679 | 0.689 | 1.000 |  |  |  | 0.8726 | 0.8222 | 0.4053 | 0.9163 |
|  |  |  |  |  |  |  |  |  |  |  | <.001 | <.001 | <.001 | <.001 | - |  |  |  |  |  |  |  |
| CRB6. You avoid crowded places (public transport, places of worship, wedding ceremony, baptism, etc.) | 0.896 | 0.868 | 0.0002 | 1 | (0-2) | 0.7701 | 0.3778 | 0.1671 | 0.2140 | 0.1905 | 0.496 | 0.534 | 0.674 | 0.671 | 0.838 | 1.000 |  |  | 0.8748 | 0.8265 | 0.4072 | 0.9161 |
|  |  |  |  |  |  |  |  |  |  |  | <.001 | <.001 | <.001 | <.001 | <.001 | - |  |  |  |  |  |  |
| CRB7. You have cut down on visits to your family and friends | 0.729 | 0.857 | <.0001 | 0 | (0-2) | 0.8175 | 0.3071 | 0.1715 | -0.0255 | 0.2074 | 0.453 | 0.499 | 0.674 | 0.666 | 0.748 | 0.771 | 1.000 |  | 0.8624 | 0.8114 | 0.4112 | 0.9173 |
|  |  |  |  |  |  |  |  |  |  |  | <.001 | <.001 | <.001 | <.001 | <.001 | <.001 | - |  |  |  |  |  |
| CRB8. You have reduced physical contacts with other members of your household | 0.671 | 0.847 | <.0001 | 0 | (0-2) | 0.7673 | 0.3135 | 0.1397 | -0.0791 | 0.2871 | 0.432 | 0.495 | 0.610 | 0.642 | 0.678 | 0.706 | 0.794 | 1.000 | 0.8243 | 0.7620 | 0.4211 | 0.9212 |
|  |  |  |  |  |  |  |  |  |  |  | <.001 | <.001 | <.001 | <.001 | <.001 | <.001 | <.001 | - |  |  |  |  |
|  |  |  |  |  |  |  |  |  |  |  |  |  |  |  |  |  |  |  |  |  | 0.4254 | 0.9307 |

| **COVID-19 Vaccination Attitudes** | **Mean** | **Standard deviation** | $\boldsymbol{p}$**-value (**$\boldsymbol{H}_{\boldsymbol{0}}\boldsymbol{:}\overline{\boldsymbol{x}}\boldsymbol{=3}$**)** | **Median** | **Range** | **Factor 1** | **Factor 2** | **Uniqueness** | **1.** | **2.** | **3.** | **4.** | **5.** | **6.** | **7.** | **8.** | **Item-test correlation** | **Item-rest correlation** | **Inter-item correlation** | **Cronbach's** $\boldsymbol{\alpha}$ **if item removed** |
| --- | --- | --- | --- | --- | --- | --- | --- | --- | --- | --- | --- | --- | --- | --- | --- | --- | --- | --- | --- | --- |
| VAC1. If a vaccine against COVID-19 was offered to you for free today, would you accept to be vaccinated? (from “I would definitively not accept” to “I would definitively accept”) | 2.744 | 1.499 | <.0001 | 3 | (1-5) | 0.8369 | -0.1358 | 0.2811 | 1.000 |  |  |  |  |  |  |  | 0.8669 | 0.8201 | 1.4411 | 0.9514 |
|  |  |  |  |  |  |  |  |  | - |  |  |  |  |  |  |  |  |  |  |  |
| VAC2. When the vaccine against COVID-19 will be widely available and free of charge in Burkina Faso: (from “I will get vaccinated as soon as possible” to “I will not be vaccinated”) | 2.761 | 1.553 | <.0001 | 3 | (1-5) | 0.8836 | -0.1483 | 0.1973 | 0.773 | 1.000 |  |  |  |  |  |  | 0.8993 | 0.8610 | 1.4034 | 0.9489 |
|  |  |  |  |  |  |  |  |  | <.001 | - |  |  |  |  |  |  |  |  |  |  |
| VAC3. Regarding vaccination against COVID-19 I would describe my attitude as: (from “Very positive” to “Very negative”) | 3.082 | 1.186 | 0.0289 | 3 | (1-5) | 0.7652 | 0.0661 | 0.4102 | 0.636 | 0.674 | 1.000 |  |  |  |  |  | 0.8000 | 0.7495 | 1.5663 | 0.9555 |
|  |  |  |  |  |  |  |  |  | <.001 | <.001 | - |  |  |  |  |  |  |  |  |  |
| VAC4. If the COVID-19 was available at the local health center, doctor's office, or pharmacy, I would: (from “Get vaccinated as soon as possible” to “I would never get vaccinated”) | 2.892 | 1.589 | 0.0317 | 3 | (1-5) | 0.9278 | -0.1055 | 0.1281 | 0.790 | 0.850 | 0.687 | 1.000 |  |  |  |  | 0.9307 | 0.9028 | 1.3691 | 0.9460 |
|  |  |  |  |  |  |  |  |  | <.001 | <.001 | <.001 | - |  |  |  |  |  |  |  |  |
| VAC5. If any of my family members or friends were considering being vaccinated against COVID-19: (from “I would strongly encourage them” to “Suggest that they do not get vaccinated”) | 2.909 | 1.295 | 0.0265 | 3 | (1-5) | 0.8309 | 0.1527 | 0.2862 | 0.662 | 0.708 | 0.646 | 0.759 | 1.000 |  |  |  | 0.8535 | 0.8108 | 1.5052 | 0.9519 |
|  |  |  |  |  |  |  |  |  | <.001 | <.001 | <.001 | <.001 | - |  |  |  |  |  |  |  |
| VAC6. I would describe myself as: (from “Looking forward to getting vaccinated against COVID-19” to “Anti-vaccination COVID-19”) | 2.770 | 1.367 | <.0001 | 3 | (1-5) | 0.8479 | 0.1104 | 0.2688 | 0.686 | 0.723 | 0.666 | 0.774 | 0.738 | 1.000 |  |  | 0.8653 | 0.8229 | 1.4772 | 0.9510 |
|  |  |  |  |  |  |  |  |  | <.001 | <.001 | <.001 | <.001 | <.001 | - |  |  |  |  |  |  |
| VAC7. Getting vaccinated against COVID-19 is: (from “Very important” to “Really irrelevant”) | 3.111 | 1.270 | 0.0062 | 3 | (1-5) | 0.8513 | 0.0933 | 0.2665 | 0.709 | 0.736 | 0.662 | 0.769 | 0.739 | 0.722 | 1.000 |  | 0.8682 | 0.8301 | 1.5026 | 0.9508 |
|  |  |  |  |  |  |  |  |  | <.001 | <.001 | <.001 | <.001 | <.001 | <.001 | - |  |  |  |  |  |
| VAC8. If I have children (or if I had children): (from “I will definitively have them vaccinated against COVID-19” to “I will definitively not vaccinate them against COVID-19”) | 2.833 | 1.482 | 0.0004 | 3 | (1-5) | 0.9144 | -0.0087 | 0.1639 | 0.782 | 0.798 | 0.699 | 0.859 | 0.760 | 0.775 | 0.784 | 1.000 | 0.9222 | 0.8941 | 1.4061 | 0.9464 |
|  |  |  |  |  |  |  |  |  | <.001 | <.001 | <.001 | <.001 | <.001 | <.001 | <.001 | - |  |  |  |  |
|  |  |  |  |  |  |  |  |  |  |  |  |  |  |  |  |  |  |  | 1.4589 | 0.9563 |

| **COVID-19 Agreement with Sanitary Measures** | **Mean** | **Standard deviation** | $\boldsymbol{p}$**-value (**$\boldsymbol{H}_{\boldsymbol{0}}\boldsymbol{:}\overline{\boldsymbol{x}}\boldsymbol{=1}$**)** | **Median** | **Range** | **Factor 1** | **Factor 2** | **Factor 3** | **Factor 4** | **Uniqueness** | **1.** | **2.** | **3.** | **4.** | **5.** | **6.** | **7.** | **8.** | **Item-test correlation** | **Item-rest correlation** | **Inter-item correlation** | **Cronbach's** $\boldsymbol{\alpha}$ **if item removed** |
| --- | --- | --- | --- | --- | --- | --- | --- | --- | --- | --- | --- | --- | --- | --- | --- | --- | --- | --- | --- | --- | --- | --- |
| ME1. The closure of schools and universities | 1.056 | 0.929 | 0.0596 | 1 | (0-2) | 0.3551 | 0.6663 | 0.4541 | -0.0295 | 0.2227 | 1.000 |  |  |  |  |  |  |  | 0.8528 | 0.7988 | 0.5548 | 0.9316 |
|  |  |  |  |  |  |  |  |  |  |  | - |  |  |  |  |  |  |  |  |  |  |  |
| ME2. The closure of places of worship | 0.883 | 0.916 | 0.0001 | 1 | (0-2) | 0.4630 | 0.6204 | 0.3082 | 0.0374 | 0.3043 | 0.745 | 1.000 |  |  |  |  |  |  | 0.8297 | 0.7717 | 0.5638 | 0.9339 |
|  |  |  |  |  |  |  |  |  |  |  | <.001 | - |  |  |  |  |  |  |  |  |  |  |
| ME3. The closure of non-essential shops (bars, non-food, and health shops, etc.) | 1.067 | 0.930 | 0.0240 | 1 | (0-2) | 0.4071 | 0.5789 | 0.4952 | 0.0120 | 0.2539 | 0.760 | 0.705 | 1.000 |  |  |  |  |  | 0.8653 | 0.8165 | 0.5518 | 0.9306 |
|  |  |  |  |  |  |  |  |  |  |  | <.001 | <.001 | - |  |  |  |  |  |  |  |  |  |
| ME4. The introduction of a curfew and movement control by the police, gendarmerie, and army | 1.052 | 0.930 | 0.0801 | 1 | (0-2) | 0.5430 | 0.3690 | 0.5337 | 0.0850 | 0.2770 | 0.636 | 0.665 | 0.706 | 1.000 |  |  |  |  | 0.8568 | 0.8062 | 0.5543 | 0.9315 |
|  |  |  |  |  |  |  |  |  |  |  | <.001 | <.001 | <.001 | - |  |  |  |  |  |  |  |  |
| ME5. The introduction of mandatory medical checks and quarantine for people entering Burkina Faso | 1.380 | 0.860 | <.0001 | 2 | (0-2) | 0.2831 | 0.3068 | 0.6379 | 0.0061 | 0.4188 | 0.591 | 0.488 | 0.601 | 0.649 | 1.000 |  |  |  | 0.7433 | 0.6689 | 0.5946 | 0.9406 |
|  |  |  |  |  |  |  |  |  |  |  | <.001 | <.001 | <.001 | <.001 | - |  |  |  |  |  |  |  |
| ME6. The closure of Burkina Faso's borders to the movement of people | 1.075 | 0.934 | 0.0116 | 1 | (0-2) | 0.4759 | 0.4352 | 0.5760 | -0.0665 | 0.2478 | 0.729 | 0.649 | 0.720 | 0.694 | 0.664 | 1.000 |  |  | 0.8733 | 0.8280 | 0.5491 | 0.9300 |
|  |  |  |  |  |  |  |  |  |  |  | <.001 | <.001 | <.001 | <.001 | <.001 | - |  |  |  |  |  |  |
| ME7. General confinement of the population with a ban on leaving the home (except for medical reasons and food) | 0.890 | 0.925 | 0.0002 | 1 | (0-2) | 0.7258 | 0.3159 | 0.3188 | -0.0054 | 0.2717 | 0.604 | 0.635 | 0.606 | 0.693 | 0.498 | 0.681 | 1.000 |  | 0.8205 | 0.7598 | 0.5647 | 0.9347 |
|  |  |  |  |  |  |  |  |  |  |  | <.001 | <.001 | <.001 | <.001 | <.001 | <.001 | - |  |  |  |  |  |
| ME8. The closure of all non-essential businesses and institutions | 0.904 | 0.917 | 0.0011 | 1 | (0-2) | 0.7123 | 0.4083 | 0.3508 | -0.0119 | 0.2027 | 0.674 | 0.677 | 0.713 | 0.717 | 0.527 | 0.727 | 0.793 | 1.000 | 0.8671 | 0.8204 | 0.5532 | 0.9304 |
|  |  |  |  |  |  |  |  |  |  |  | <.001 | <.001 | <.001 | <.001 | <.001 | <.001 | <.001 | - |  |  |  |  |
|  |  |  |  |  |  |  |  |  |  |  |  |  |  |  |  |  |  |  |  |  | 0.5608 | 0.9409 |

| **COVID-19 Conspiracy Beliefs** | **Mean** | **Standard deviation** | $\boldsymbol{p}$**-value (**$\boldsymbol{H}_{\boldsymbol{0}}\boldsymbol{:}\overline{\boldsymbol{x}}\boldsymbol{=3}$**)** | **Median** | **Range** | **Factor 1** | **Factor 2** | **Factor 3** | **Factor 4** | **Uniqueness** | **1.** | **2.** | **3.** | **4.** | **5.** | **6.** | **7.** | **Item-test correlation** | **Item-rest correlation** | **Inter-item correlation** | **Cronbach's** $\boldsymbol{\alpha}$ **if item removed** |
| --- | --- | --- | --- | --- | --- | --- | --- | --- | --- | --- | --- | --- | --- | --- | --- | --- | --- | --- | --- | --- | --- |
| CB1. Government withholds important information from the population on COVID-19 outbreak | 3.129 | 1.109 | 0.0002 | 3 | (1-5) | 0.7715 | 0.2685 | 0.2759 | -0.0159 | 0.2564 | 1.000 |  |  |  |  |  |  | 0.7984 | 0.7186 | 0.7512 | 0.9093 |
|  |  |  |  |  |  |  |  |  |  |  | - |  |  |  |  |  |  |  |  |  |  |
| CB2. Doctors and scientists hide important information from the population on the COVID-19 epidemic | 3.020 | 1.066 | 0.5532 | 3 | (1-5) | 0.7883 | 0.2827 | 0.2367 | 0.0140 | 0.2424 | 0.806 | 1.000 |  |  |  |  |  | 0.7903 | 0.7117 | 0.7628 | 0.9099 |
|  |  |  |  |  |  |  |  |  |  |  | <.001 | - |  |  |  |  |  |  |  |  |  |
| CB3. The COVID-19 epidemic is a deliberate attempt to reduce the population size of poor countries | 2.865 | 1.044 | <.0001 | 3 | (1-5) | 0.4251 | 0.5973 | 0.4247 | 0.0456 | 0.2801 | 0.577 | 0.633 | 1.000 |  |  |  |  | 0.8524 | 0.7952 | 0.7395 | 0.9016 |
|  |  |  |  |  |  |  |  |  |  |  | <.001 | <.001 | - |  |  |  |  |  |  |  |  |
| CB4. The COVID-19 virus is intentionally presented as dangerous to manipulate the public | 2.856 | 1.069 | <.0001 | 3 | (1-5) | 0.3888 | 0.6519 | 0.3327 | -0.0078 | 0.3132 | 0.576 | 0.567 | 0.711 | 1.000 |  |  |  | 0.8234 | 0.7551 | 0.7476 | 0.9056 |
|  |  |  |  |  |  |  |  |  |  |  | <.001 | <.001 | <.001 | - |  |  |  |  |  |  |  |
| CB5. The COVID-19 epidemic is part of a global effort to impose mandatory vaccination | 2.954 | 1.080 | 0.1781 | 3 | (1-5) | 0.3206 | 0.6025 | 0.4956 | -0.0353 | 0.2874 | 0.557 | 0.520 | 0.701 | 0.729 | 1.000 |  |  | 0.8418 | 0.7788 | 0.7370 | 0.9032 |
|  |  |  |  |  |  |  |  |  |  |  | <.001 | <.001 | <.001 | <.001 | - |  |  |  |  |  |  |
| CB6. Pharmaceutical industry promotes the spread of the COVID-19 to make money | 3.060 | 1.148 | 0.0987 | 3 | (1-5) | 0.3403 | 0.3199 | 0.6552 | -0.0151 | 0.3523 | 0.541 | 0.516 | 0.590 | 0.536 | 0.658 | 1.000 |  | 0.8001 | 0.7174 | 0.7431 | 0.9097 |
|  |  |  |  |  |  |  |  |  |  |  | <.001 | <.001 | <.001 | <.001 | <.001 | - |  |  |  |  |  |
| CB7. The COVID-19 pandemic is a deliberate attempt by rich country governments to better control the populations of poor countries | 2.950 | 1.161 | 0.1737 | 3 | (1-5) | 0.3072 | 0.4448 | 0.6518 | 0.0219 | 0.2824 | 0.533 | 0.513 | 0.704 | 0.627 | 0.676 | 0.724 | 1.000 | 0.8364 | 0.7654 | 0.7230 | 0.9046 |
|  |  |  |  |  |  |  |  |  |  |  | <.001 | <.001 | <.001 | <.001 | <.001 | <.001 | - |  |  |  |  |
|  |  |  |  |  |  |  |  |  |  |  |  |  |  |  |  |  |  |  |  | 0.7434 | 0.9186 |

| **Institutional Mistrust** | **Mean** | **Standard deviation** | $\boldsymbol{p}$**-value (**$\boldsymbol{H}_{\boldsymbol{0}}\boldsymbol{:}\overline{\boldsymbol{x}}\boldsymbol{=3}$**)** | **Median** | **Range** | **Factor 1** | **Factor 2** | **Factor 3** | **Uniqueness** | **1.** | **2.** | **3.** | **4.** | **5.** | **6.** | **7.** | **Item-test correlation** | **Item-rest correlation** | **Inter-item correlation** | **Cronbach's** $\boldsymbol{\alpha}$ **if item removed** |
| --- | --- | --- | --- | --- | --- | --- | --- | --- | --- | --- | --- | --- | --- | --- | --- | --- | --- | --- | --- | --- |
| MIS1. Do you trust the President of the Republic (Roch Marc Christian Kaboré)? | 2.231 | 1.322 | <.0001 | 2 | (1-5) | 0.7487 | 0.2297 | 0.2428 | 0.3277 | 1.000 |  |  |  |  |  |  | 0.7650 | 0.6498 | 0.6139 | 0.8096 |
|  |  |  |  |  |  |  |  |  |  | - |  |  |  |  |  |  |  |  |  |  |
| MIS2. Do you trust the government? | 2.548 | 1.392 | <.0001 | 2 | (1-5) | 0.7856 | 0.2426 | 0.1966 | 0.2853 | 0.758 | 1.000 |  |  |  |  |  | 0.7725 | 0.6522 | 0.6007 | 0.8096 |
|  |  |  |  |  |  |  |  |  |  | <.001 | - |  |  |  |  |  |  |  |  |  |
| MIS3. Do you trust the local authorities(municipal/regional councilors, mayors, prefects, etc.)? | 2.300 | 1.368 | <.0001 | 2 | (1-5) | 0.4706 | 0.5042 | 0.2193 | 0.4762 | 0.500 | 0.574 | 1.000 |  |  |  |  | 0.7703 | 0.6520 | 0.6051 | 0.8095 |
|  |  |  |  |  |  |  |  |  |  | <.001 | <.001 | - |  |  |  |  |  |  |  |  |
| MIS4. Do you trust the traditional authorities (neighborhood chief, village/canton chief, etc.)? | 1.722 | 1.070 | <.0001 | 1 | (1-5) | 0.2415 | 0.7505 | 0.1734 | 0.3483 | 0.400 | 0.400 | 0.562 | 1.000 |  |  |  | 0.7072 | 0.6024 | 0.6789 | 0.8187 |
|  |  |  |  |  |  |  |  |  |  | <.001 | <.001 | <.001 | - |  |  |  |  |  |  |  |
| MIS5. Do you trust the religious authorities (religious leader, etc.)? | 1.608 | 1.003 | <.0001 | 1 | (1-5) | 0.1980 | 0.7200 | 0.2561 | 0.3767 | 0.378 | 0.376 | 0.504 | 0.711 | 1.000 |  |  | 0.7027 | 0.6046 | 0.6906 | 0.8194 |
|  |  |  |  |  |  |  |  |  |  | <.001 | <.001 | <.001 | <.001 | - |  |  |  |  |  |  |
| MIS6. Do you trust the scientific authorities (doctors, other health professionals, researchers, etc.)? | 2.044 | 1.143 | <.0001 | 2 | (1-5) | 0.2098 | 0.2551 | 0.6770 | 0.4326 | 0.399 | 0.333 | 0.391 | 0.351 | 0.412 | 1.000 |  | 0.6865 | 0.5672 | 0.6777 | 0.8227 |
|  |  |  |  |  |  |  |  |  |  | <.001 | <.001 | <.001 | <.001 | <.001 | - |  |  |  |  |  |
| MIS7. Do you trust the World Health Organization (WHO)? | 2.392 | 1.270 | <.0001 | 2 | (1-5) | 0.1752 | 0.1485 | 0.6577 | 0.5148 | 0.311 | 0.312 | 0.289 | 0.254 | 0.307 | 0.612 | 1.000 | 0.6136 | 0.4584 | 0.7000 | 0.8395 |
|  |  |  |  |  |  |  |  |  |  | <.001 | <.001 | <.001 | <.001 | <.001 | <.001 | - |  |  |  |  |
|  |  |  |  |  |  |  |  |  |  |  |  |  |  |  |  |  |  |  | 0.6524 | 0.8406 |

# **Supplementary File 5 – Complete structural equations models with specifications**

| **Variables** | **Containment-related Behaviors** | | | | | |
| --- | --- | --- | --- | --- | --- | --- |
|  | **Standardized Coefficient** | **Standard Error** | $\boldsymbol{z}$**-value** | $\boldsymbol{p}$**-value** | **95% Confidence Interval** | |
| **Containment-related Behaviors** |  |  |  |  |  |  |
| Institutional Mistrust | **-0.206***** | 0.042 | -4.860 | **<.001** | -0.289 | -0.123 |
| COVID-19 Conspiracy Beliefs | **-0.158***** | 0.037 | -4.290 | **<.001** | -0.231 | -0.086 |
| Gender (ref: male) | **0.085*** | 0.034 | 2.520 | **0.012** | 0.019 | 0.151 |
| Age (in years) | **0.121**** | 0.037 | 3.300 | **0.001** | 0.049 | 0.192 |
| Attended School (ref: no) | **0.077*** | 0.038 | 2.030 | **0.042** | 0.003 | 0.151 |
| Live with a Partner (ref: no) | **0.135***** | 0.037 | 3.650 | **<.001** | 0.062 | 0.207 |
| Salaried Employment (ref: no) | **0.201***** | 0.035 | 5.790 | **<.001** | 0.133 | 0.269 |
| Urban residency (ref: rural) | **-0.079*** | 0.036 | -2.190 | **0.028** | -0.149 | -0.008 |
| Self-employed (ref: no) | 0.059 | 0.034 | 1.730 | 0.083 | -0.008 | 0.126 |
| **Institutional Mistrust** |  |  |  |  |  |  |
| Traditional Information Seeking Frequency Score | -0.002 | 0.043 | -0.050 | 0.957 | -0.086 | 0.082 |
| Digital Information Seeking Frequency Score | 0.084 | 0.048 | 1.760 | 0.079 | -0.010 | 0.177 |
| COVID-19 Negative Sentiments Score | **0.126***** | 0.034 | 3.720 | **<.001** | 0.060 | 0.193 |
| Gender (ref: male) | -0.036 | 0.035 | -1.010 | 0.312 | -0.105 | 0.034 |
| Age (in years) | 0.025 | 0.039 | 0.660 | 0.511 | -0.050 | 0.101 |
| Attended School (ref: no) | **0.144***** | 0.041 | 3.480 | **<.001** | 0.063 | 0.225 |
| Live with a Partner (ref: no) | **0.158***** | 0.038 | 4.220 | **<.001** | 0.085 | 0.232 |
| Salaried Employment (ref: no) | 0.063 | 0.039 | 1.620 | 0.106 | -0.013 | 0.139 |
| Urban residency (ref: rural) | **0.202***** | 0.035 | 5.720 | **<.001** | 0.133 | 0.272 |
| Self-employed (ref: no) | **0.075*** | 0.035 | 2.130 | **0.033** | 0.006 | 0.144 |
| **COVID-19 Conspiracy Beliefs** |  |  |  |  |  |  |
| Institutional Mistrust | **0.288***** | 0.040 | 7.210 | **<.001** | 0.210 | 0.366 |
| Traditional Information Seeking Frequency Score | **0.124**** | 0.041 | 3.000 | **0.003** | 0.043 | 0.205 |
| Digital Information Seeking Frequency Score | -0.044 | 0.046 | -0.950 | 0.342 | -0.135 | 0.047 |
| COVID-19 Negative Sentiments Score | 0.005 | 0.034 | 0.140 | 0.887 | -0.061 | 0.071 |
| Gender (ref: male) | 0.034 | 0.034 | 0.980 | 0.326 | -0.034 | 0.101 |
| Age (in years) | -0.023 | 0.037 | -0.610 | 0.539 | -0.096 | 0.050 |
| Attended School (ref: no) | 0.052 | 0.041 | 1.270 | 0.203 | -0.028 | 0.131 |
| Live with a Partner (ref: no) | 0.011 | 0.037 | 0.290 | 0.775 | -0.063 | 0.084 |
| Salaried Employment (ref: no) | -0.010 | 0.038 | -0.260 | 0.796 | -0.083 | 0.064 |
| Urban residency (ref: rural) | 0.061 | 0.036 | 1.700 | 0.090 | -0.009 | 0.131 |
| Self-employed (ref: no) | 0.044 | 0.034 | 1.280 | 0.199 | -0.023 | 0.111 |
| **COVID-19 Negative Sentiments Score** |  |  |  |  |  |  |
| Traditional Information Seeking Frequency Score | -0.007 | 0.041 | -0.170 | 0.861 | -0.088 | 0.074 |
| Digital Information Seeking Frequency Score | -0.089 | 0.046 | -1.950 | 0.051 | -0.179 | 0.000 |
| Gender (ref: male) | **-0.076*** | 0.034 | -2.250 | **0.024** | -0.143 | -0.010 |
| Age (in years) | 0.017 | 0.037 | 0.460 | 0.649 | -0.056 | 0.090 |
| Attended School (ref: no) | 0.025 | 0.040 | 0.630 | 0.529 | -0.053 | 0.103 |
| Live with a Partner (ref: no) | **0.078*** | 0.036 | 2.160 | **0.031** | 0.007 | 0.150 |
| Salaried Employment (ref: no) | **-0.100**** | 0.037 | -2.700 | **0.007** | -0.172 | -0.027 |
| Urban residency (ref: rural) | 0.028 | 0.035 | 0.790 | 0.428 | -0.040 | 0.096 |
| Self-employed (ref: no) | **-0.067*** | 0.034 | -1.980 | **0.048** | -0.133 | -0.001 |
| Constant | **5.562***** | 0.202 | 27.540 | **<.001** | 5.166 | 5.958 |
| Number of observations | 925 | | | | | |
| Number of parameters | 115 | | | | | |
| Log Likelihood | -34,757.323 | | | | | |
| AIC | 69,744.647 | | | | | |
| AIC3 | 69,859.647 | | | | | |
| CAIC | 70,415.073 | | | | | |
| BIC | 70,300.073 | | | | | |
| HBIC | 70,088.717 | | | | | |
| ABIC | 69,547.383 | | | | | |
| RMSEA | 0.047 | | | | | |
|  | [0.044, 0.050] | | | | | |
| SRMR | 0.043 | | | | | |
| CFI | 0.941 | | | | | |
| TLI | 0.931 | | | | | |
| $\chi^{2}$ | 1195.597 | | | | | |
| $p$-value | <.001 | | | | | |

*p<0.05, ** p<0.01, *** p<0.001

| **Variables** | **Vaccination Attitudes** | | | | | |
| --- | --- | --- | --- | --- | --- | --- |
|  | **Standardized Coefficient** | **Standard Error** | $\boldsymbol{z}$**-value** | $\boldsymbol{p}$**-value** | **95% Confidence Interval** | |
| **Vaccination Attitudes** |  |  |  |  |  |  |
| Institutional Mistrust | **-0.228***** | 0.036 | -6.240 | **<.001** | -0.299 | -0.156 |
| COVID-19 Conspiracy Beliefs | **-0.412***** | 0.030 | -13.950 | **<.001** | -0.470 | -0.354 |
| Gender (ref: male) | **0.113***** | 0.029 | 3.950 | **<.001** | 0.057 | 0.169 |
| Age (in years) | **0.074*** | 0.031 | 2.390 | **0.017** | 0.013 | 0.135 |
| Attended School (ref: no) | -0.028 | 0.032 | -0.860 | 0.389 | -0.090 | 0.035 |
| Live with a Partner (ref: no) | -0.030 | 0.031 | -0.950 | 0.340 | -0.092 | 0.032 |
| Salaried Employment (ref: no) | -0.005 | 0.030 | -0.160 | 0.872 | -0.064 | 0.054 |
| Urban residency (ref: rural) | **-0.061*** | 0.030 | -2.020 | **0.044** | -0.121 | -0.002 |
| Self-employed (ref: no) | -0.018 | 0.029 | -0.620 | 0.532 | -0.075 | 0.039 |
| **Institutional Mistrust** |  |  |  |  |  |  |
| Traditional Information Seeking Frequency Score | -0.011 | 0.043 | -0.250 | 0.805 | -0.095 | 0.074 |
| Digital Information Seeking Frequency Score | 0.093 | 0.048 | 1.950 | 0.052 | -0.001 | 0.187 |
| COVID-19 Negative Sentiments Score | **0.128***** | 0.034 | 3.750 | **<.001** | 0.061 | 0.195 |
| Gender (ref: male) | -0.029 | 0.036 | -0.800 | 0.425 | -0.099 | 0.042 |
| Age (in years) | 0.022 | 0.039 | 0.580 | 0.565 | -0.054 | 0.098 |
| Attended School (ref: no) | **0.145***** | 0.041 | 3.510 | **<.001** | 0.064 | 0.226 |
| Live with a Partner (ref: no) | **0.149***** | 0.038 | 3.960 | **<.001** | 0.075 | 0.223 |
| Salaried Employment (ref: no) | 0.066 | 0.039 | 1.690 | 0.091 | -0.010 | 0.142 |
| Urban residency (ref: rural) | **0.201***** | 0.035 | 5.680 | **<.001** | 0.132 | 0.271 |
| Self-employed (ref: no) | **0.077*** | 0.035 | 2.180 | **0.029** | 0.008 | 0.146 |
| **COVID-19 Conspiracy Beliefs** |  |  |  |  |  |  |
| Institutional Mistrust | **0.283***** | 0.040 | 7.090 | **<.001** | 0.205 | 0.362 |
| Traditional Information Seeking Frequency Score | **0.127**** | 0.041 | 3.060 | **0.002** | 0.046 | 0.208 |
| Digital Information Seeking Frequency Score | -0.037 | 0.046 | -0.790 | 0.431 | -0.128 | 0.055 |
| COVID-19 Negative Sentiments Score | 0.007 | 0.034 | 0.210 | 0.831 | -0.059 | 0.073 |
| Gender (ref: male) | 0.041 | 0.034 | 1.180 | 0.237 | -0.027 | 0.108 |
| Age (in years) | -0.015 | 0.037 | -0.400 | 0.690 | -0.088 | 0.058 |
| Attended School (ref: no) | 0.044 | 0.040 | 1.090 | 0.275 | -0.035 | 0.124 |
| Live with a Partner (ref: no) | 0.011 | 0.037 | 0.310 | 0.758 | -0.061 | 0.084 |
| Salaried Employment (ref: no) | -0.004 | 0.038 | -0.110 | 0.910 | -0.078 | 0.069 |
| Urban residency (ref: rural) | 0.057 | 0.036 | 1.600 | 0.109 | -0.013 | 0.128 |
| Self-employed (ref: no) | 0.046 | 0.034 | 1.340 | 0.179 | -0.021 | 0.113 |
| **COVID-19 Negative Sentiments Score** |  |  |  |  |  |  |
| Traditional Information Seeking Frequency Score | -0.020 | 0.041 | -0.480 | 0.630 | -0.101 | 0.061 |
| Digital Information Seeking Frequency Score | -0.071 | 0.046 | -1.540 | 0.123 | -0.161 | 0.019 |
| Gender (ref: male) | **-0.079*** | 0.034 | -2.310 | **0.021** | -0.145 | -0.012 |
| Age (in years) | 0.005 | 0.037 | 0.130 | 0.898 | -0.068 | 0.077 |
| Attended School (ref: no) | 0.016 | 0.040 | 0.410 | 0.685 | -0.062 | 0.094 |
| Live with a Partner (ref: no) | 0.073 | 0.036 | 2.010 | 0.045 | 0.002 | 0.144 |
| Salaried Employment (ref: no) | **-0.107**** | 0.037 | -2.890 | **0.004** | -0.179 | -0.034 |
| Urban residency (ref: rural) | 0.046 | 0.035 | 1.320 | 0.185 | -0.022 | 0.114 |
| Self-employed (ref: no) | **-0.070*** | 0.034 | -2.080 | **0.037** | -0.136 | -0.004 |
| Constant | **5.576***** | 0.200 | 27.940 | **<.001** | 5.185 | 5.967 |
| Number of observations | 925 | | | | | |
| Number of parameters | 112 | | | | | |
| Log Likelihood | -37,165.594 | | | | | |
| AIC | 74,555.187 | | | | | |
| AIC3 | 74,667.187 | | | | | |
| CAIC | 75,208.124 | | | | | |
| BIC | 75,096.124 | | | | | |
| HBIC | 74,890.282 | | | | | |
| ABIC | 74,363.070 | | | | | |
| RMSEA | 0.046 | | | | | |
|  | [0.043, 0.049] | | | | | |
| SRMR | 0.040 | | | | | |
| CFI | 0.952 | | | | | |
| TLI | 0.944 | | | | | |
| $\chi^{2}$ | 1169.449 | | | | | |
| $p$-value | <.001 | | | | | |

*p<0.05, ** p<0.01, *** p<0.001

| **Variables** | **Agreement with sanitary measures** | | | | | |
| --- | --- | --- | --- | --- | --- | --- |
|  | **Standardized Coefficient** | **Standard Error** | $\boldsymbol{z}$**-value** | $\boldsymbol{p}$**-value** | **95% Confidence Interval** | |
| **Agreement with sanitary measures** |  |  |  |  |  |  |
| Institutional Mistrust | **-0.294***** | 0.042 | -7.040 | **<.001** | -0.376 | -0.212 |
| COVID-19 Conspiracy Beliefs | **-0.129***** | 0.037 | -3.480 | **<.001** | -0.201 | -0.056 |
| Gender (ref: male) | **0.097**** | 0.033 | 2.920 | **0.004** | 0.032 | 0.163 |
| Age (in years) | 0.040 | 0.036 | 1.100 | 0.271 | -0.031 | 0.111 |
| Attended School (ref: no) | -0.037 | 0.037 | -0.990 | 0.324 | -0.110 | 0.037 |
| Live with a Partner (ref: no) | -0.027 | 0.037 | -0.730 | 0.464 | -0.099 | 0.045 |
| Salaried Employment (ref: no) | **0.106**** | 0.035 | 3.030 | **0.002** | 0.038 | 0.175 |
| Urban residency (ref: rural) | -0.037 | 0.035 | -1.040 | 0.301 | -0.106 | 0.033 |
| Self-employed (ref: no) | -0.064 | 0.034 | -1.910 | 0.056 | -0.130 | 0.002 |
| **Institutional Mistrust** |  |  |  |  |  |  |
| Traditional Information Seeking Frequency Score | -0.005 | 0.044 | -0.110 | 0.909 | -0.092 | 0.082 |
| Digital Information Seeking Frequency Score | 0.059 | 0.049 | 1.200 | 0.228 | -0.037 | 0.154 |
| COVID-19 Negative Sentiments Score | **0.132***** | 0.035 | 3.780 | **<.001** | 0.064 | 0.201 |
| Gender (ref: male) | -0.033 | 0.037 | -0.890 | 0.371 | -0.105 | 0.039 |
| Age (in years) | 0.013 | 0.040 | 0.330 | 0.742 | -0.065 | 0.092 |
| Attended School (ref: no) | **0.155***** | 0.043 | 3.630 | **<.001** | 0.071 | 0.239 |
| Live with a Partner (ref: no) | **0.160***** | 0.039 | 4.130 | **<.001** | 0.084 | 0.236 |
| Salaried Employment (ref: no) | 0.070 | 0.040 | 1.740 | 0.082 | -0.009 | 0.148 |
| Urban residency (ref: rural) | **0.210***** | 0.036 | 5.810 | **<.001** | 0.139 | 0.281 |
| Self-employed (ref: no) | **0.087*** | 0.036 | 2.420 | **0.015** | 0.017 | 0.158 |
| **COVID-19 Conspiracy Beliefs** |  |  |  |  |  |  |
| Institutional Mistrust | **0.304***** | 0.041 | 7.480 | **<.001** | 0.224 | 0.384 |
| Traditional Information Seeking Frequency Score | **0.150***** | 0.042 | 3.550 | **<.001** | 0.067 | 0.233 |
| Digital Information Seeking Frequency Score | -0.056 | 0.047 | -1.180 | 0.238 | -0.148 | 0.037 |
| COVID-19 Negative Sentiments Score | -0.009 | 0.035 | -0.270 | 0.789 | -0.077 | 0.058 |
| Gender (ref: male) | 0.014 | 0.035 | 0.410 | 0.683 | -0.055 | 0.084 |
| Age (in years) | -0.041 | 0.038 | -1.060 | 0.287 | -0.116 | 0.034 |
| Attended School (ref: no) | 0.031 | 0.042 | 0.740 | 0.459 | -0.051 | 0.113 |
| Live with a Partner (ref: no) | 0.005 | 0.038 | 0.130 | 0.895 | -0.070 | 0.080 |
| Salaried Employment (ref: no) | -0.024 | 0.039 | -0.630 | 0.530 | -0.100 | 0.052 |
| Urban residency (ref: rural) | **0.079*** | 0.037 | 2.140 | **0.032** | 0.007 | 0.150 |
| Self-employed (ref: no) | 0.046 | 0.035 | 1.320 | 0.188 | -0.022 | 0.115 |
| **COVID-19 Negative Sentiments Score** |  |  |  |  |  |  |
| Traditional Information Seeking Frequency Score | -0.018 | 0.043 | -0.410 | 0.679 | -0.101 | 0.066 |
| Digital Information Seeking Frequency Score | -0.084 | 0.047 | -1.800 | 0.072 | -0.176 | 0.008 |
| Gender (ref: male) | **-0.100**** | 0.035 | -2.870 | **0.004** | -0.169 | -0.032 |
| Age (in years) | 0.018 | 0.038 | 0.460 | 0.644 | -0.057 | 0.093 |
| Attended School (ref: no) | 0.017 | 0.041 | 0.400 | 0.689 | -0.064 | 0.097 |
| Live with a Partner (ref: no) | **0.081*** | 0.037 | 2.170 | **0.030** | 0.008 | 0.155 |
| Salaried Employment (ref: no) | **-0.111**** | 0.038 | -2.920 | **0.004** | -0.186 | -0.036 |
| Urban residency (ref: rural) | 0.020 | 0.036 | 0.560 | 0.575 | -0.050 | 0.090 |
| Self-employed (ref: no) | -0.063 | 0.035 | -1.830 | 0.068 | -0.131 | 0.005 |
| Constant | **5.550** | 0.207 | 26.850 | **<.001** | 5.145 | 5.955 |
| Number of observations | 871 | | | | | |
| Number of parameters | 113 | | | | | |
| Log Likelihood | -32,839.807 | | | | | |
| AIC | 65,905.615 | | | | | |
| AIC3 | 66,018.615 | | | | | |
| CAIC | 66,557.584 | | | | | |
| BIC | 66,444.584 | | | | | |
| HBIC | 66,236.904 | | | | | |
| ABIC | 65,711.499 | | | | | |
| RMSEA | 0.057 | | | | | |
|  | [0.054, 0.060] | | | | | |
| SRMR | 0.045 | | | | | |
| CFI | 0.922 | | | | | |
| TLI | 0.909 | | | | | |
| $\chi^{2}$ | 1487.927 | | | | | |
| $p$-value | <.001 | | | | | |

*p<0.05, ** p<0.01, *** p<0.001
